# Seasonal Synchronization of Influenza in the United States Older Adult Population

**DOI:** 10.1371/journal.pone.0010187

**Published:** 2010-04-15

**Authors:** Julia B. Wenger, Elena N. Naumova

**Affiliations:** Department of Public Health and Community Medicine, Tufts University School of Medicine, Boston, Massachusetts, United States of America; University of Oxford, United Kingdom

## Abstract

**Background:**

In temperate regions, influenza epidemics occur annually with the highest activity occurring during the winter months. While seasonal dynamics of the influenza virus, such as time of onset and circulating strains, are well documented by the Centers for Disease Control and Prevention Influenza Surveillance System, an accurate prediction of timing, magnitude, and composition of circulating strains of seasonal influenza remains elusive. To facilitate public health preparedness for seasonal influenza and to obtain better insights into the spatiotemporal behavior of emerging strains, it is important to develop measurable characteristics of seasonal oscillation and to quantify the relationships between those parameters on a spatial scale. The objectives of our research were to examine the seasonality of influenza on a national and state level as well as the relationship between peak timing and intensity of influenza in the United States older adult population.

**Methodology/Principal Findings:**

A total of 248,889 hospitalization records were extracted from the Centers for Medicare and Medicaid Services for the influenza seasons 1991–2004. Harmonic regression models were used to quantify the peak timing and absolute intensity for each of the 48 contiguous states and Washington, DC. We found that individual influenza seasons showed spatial synchrony with consistent late or early timing occurring across all 48 states during each influenza season in comparison to the overall average. On a national level, seasons that had an earlier peak also had higher rates of influenza (r_s_ = −0.5). We demonstrated a spatial trend in peak timing of influenza; western states such as Nevada, Utah, and California peaked earlier and New England States such as Rhode Island, Maine, and New Hampshire peaked later.

**Conclusions/Significance:**

Our findings suggest that a systematic description of influenza seasonal patterns is a valuable tool for disease surveillance and can facilitate strategies for prevention of severe disease in the vulnerable, older adult population.

## Introduction

In a temperate region such as the United States, influenza epidemics occur every year with the highest activity occurring during the winter months [1]–[3]. Regardless of its regularity, an accurate prediction of timing, magnitude, and composition of circulating strains of seasonal influenza remains elusive [4]. Influenza viruses can cause disease among any age group, however, rates of serious illness and death are highest among older adults (those aged 65 years and older) [5].While influenza-associated morbidity has declined among most age groups over the past 3 decades, hospitalizations have increased during the same time period for the older adult population [6]–[8]. Although seasonal dynamics of the influenza virus, such as time of onset and circulating strains, are well documented by the Centers for Disease Control and Prevention Influenza Surveillance System [9], there has been difficulty establishing predictive models for seasonal influenza in the susceptible older adult population. It is vital to quantify seasonal pattern of influenza, to determine reliable estimates for characterization of seasonal influenza, and to establish the relationships between those parameters for public health preparedness for influenza epidemics and pandemics [10]–[11]. The utilization of large databases of clinical records provides a unique opportunity to characterize seasonal influenza on a national scale.

The dynamics of influenza outbreaks can be characterized by periodicity, severity, and a number of other parameters useful to describing the onset, duration, and intensity of outbreaks. Peak timing is one of the essential characteristics of outbreak dynamics and it reflects the time when an outbreak reaches its maximum intensity. Although it does not directly indicate the onset of disease in a population, it reflects the primary characteristic of outbreak dynamics, and therefore contains important epidemiological information. Considering the route of human-to-human transmission of influenza, the change in peak timing in adjacent geographical areas can manifest in a form of traveling waves, a special case of spatial disease dynamics. The explicit geographical transition can be distorted by less-defined structures of mass migration. Human movement (such as by air travel) and population size have been shown to impact the timing of influenza epidemics across the United States [12]–[14]. Synchrony is another phenomenon associated with the peak timing of influenza. Spatial synchronization dictates that geographical regions which have similar patterns of annual timing of disease incidence are likely to show similar patterns in influenza movement across seasons, which is in part mediated by circulating strains [15]–[17].

Another characteristic, intensity, or the maximum seasonal incidence of influenza, is an indicator of disease severity. Seasonally, influenza varies significantly in its magnitude due to the strain virulence and host susceptibility. A severity index has been used to measure excess mortality during epidemic and non-epidemic influenza season. This severity index utilized cyclical regression to measure the intensity of baseline influenza. The researchers examining excess mortality also observed variability in intensity in relation to changes in circulating strains of influenza [18], however other factors may also play a role in viral evolution and influenza intensity [4], [19]–[20].

While each parameter can contribute a significant amount of information about individual influenza seasons, it is the unique combination of these parameters that can improve predictability of seasonal influenza epidemics. Previous research has found that seasons with high intensity or disease burden are often followed by seasons with lower intensity [21]. This cyclical pattern has been shown to be dictated, in part, to the week at which influenza is at its highest [22]. Such a pattern can be seen in the epidemic 2003/2004 influenza season where an early start to the influenza season was associated with above average morbidity and mortality [23].

As influenza follows a specific pattern of seasonal variation, seasonal oscillations can be measured by a variety of techniques [24]–[25], including non-linear time series [26]. Widely accepted, Serfling regression allows direct estimation of regression parameters, that can be further used for seasonal characterization [27]. We have developed a method to study seasonality with formal structures allowing for comparison of parameters such as peak timing and absolute intensity seasonally, both spatially and temporally [28]–[30]. This method has been applied and validated across multiple infectious diseases with well defined seasonal patterns [31]. By using this technique, a comprehensive, systematic, and detailed examination of the seasonal patterns of influenza can be made with straightforward interpretations, providing a valuable tool for biosurveillance [30].

Due to the significance of understanding spatiotemporal trends of influenza in the United States older adult population, our objectives were as follows: 1) to examine national trends of seasonality as well as the relationship between peak timing and intensity of influenza in the US older adult population, and 2) to assess seasonal variation across individual influenza seasons on a state-by-state basis and the relationship between peak timing and intensity of influenza seasons on the state level. To achieve these objectives we utilized a comprehensive source of hospitalization data, covering 98% of the United States older adult population [32].

## Methods

### Hospitalizations

**Figure 1:**
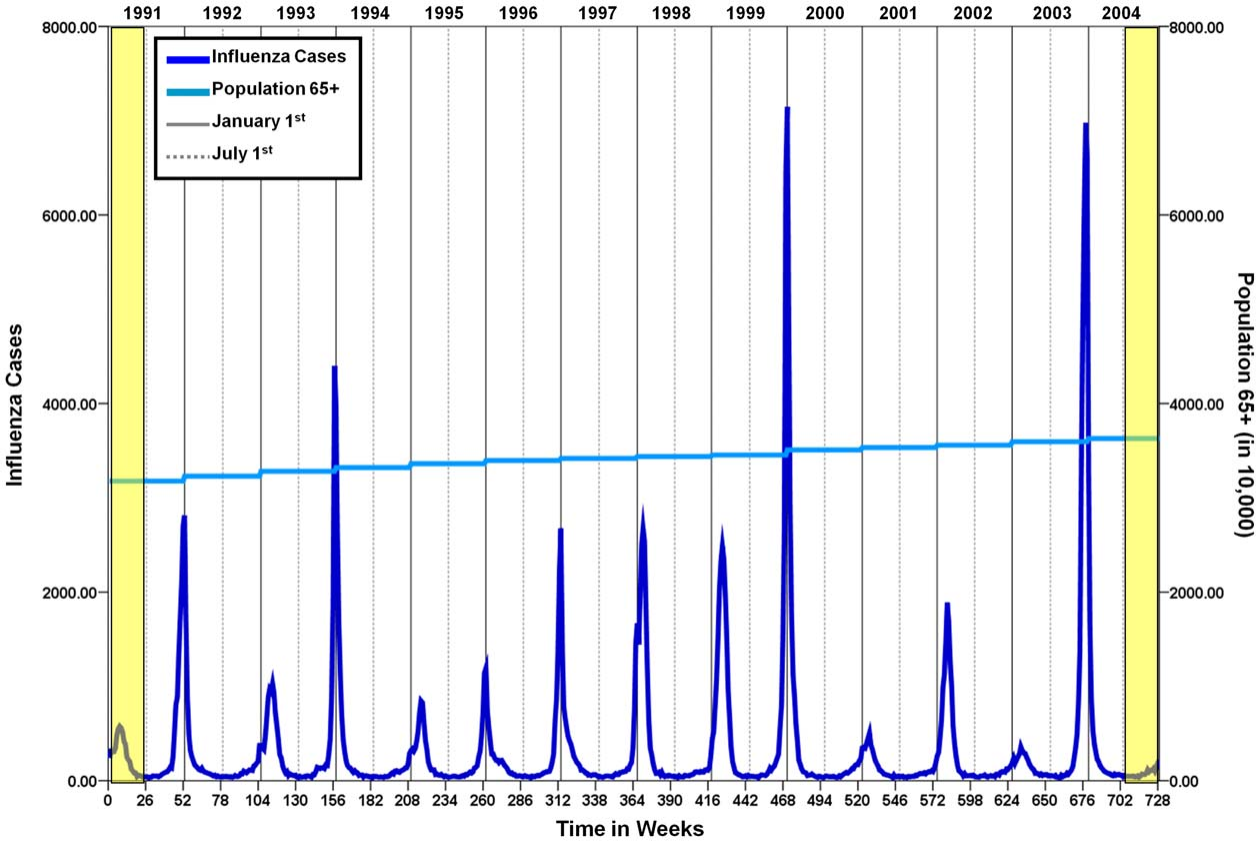
Weekly counts of influenza hospitalizations and population estimates for US older adults for 13 influenza seasons (1991–2004).

Data on hospitalization rates for influenza among older adults were abstracted from Centers for Medicare and Medicaid Services (CMS) for the years 1991 through 2004. Of the 136.2 million hospitalization records in CMS, 14.3 million (10.5%) were identified as hospitalizations for pneumonia and influenza, International Classification of Diseases, Ninth Revision, Clinical Modification Codes (ICD-9CM) 480–487. Of these, 248,889 influenza records (1.7% of pneumonia and influenza records) were analyzed. Variables available in the CMS dataset include age, gender, state of residence, date of admission, date of discharge, and up to 10 diagnostic fields. Influenza was defined using ICD-9CM 487 for any of the 10 diagnosis coding slots. Using the date of admission, we created a set of time series of weekly counts of influenza-related hospitalizations for each state and nationwide. To calculate weekly hospitalization rates, we obtained US population estimates from the US Census 1990 and 2000 and interpolated population estimates for each week of analysis. Figure 1 illustrates the weekly counts of hospitalizations and annual population estimates of the older adult population in the continental US. Yellow, highlighted bars represent the first and last 3 months of the time series that were removed for the purpose of seasonality assessment. Influenza hospitalizations for each state and each week were divided by the interpolated annual population counts for the older adult population. Thirteen influenza seasons based on date of admission, defined as July 1^st^ through June 30^th^ of the subsequent calendar year, were examined for similarity in seasonal characteristics for each of the 48 contiguous states and Washington, DC (subsequently referred to as 48 states).

### Seasonal Characteristics

**Figure 2:**
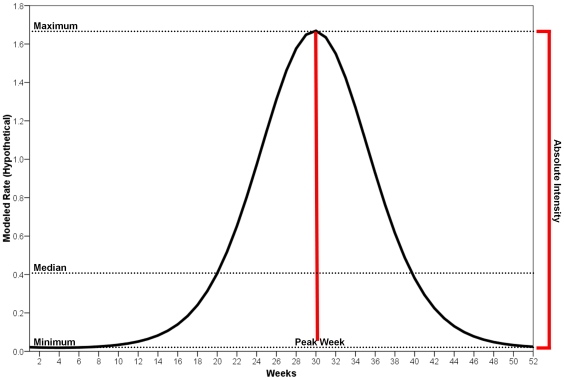
Hypothetical annual harmonic regression curve and its attributes.

The seasonal patterns of influenza-related hospitalization records were analyzed with a harmonic regression model adapted for a Poisson-distribution outcome [33]. We expanded the utility of this model by estimating the essential parameters using the δ-method [30]. Specifically, we estimated peak timing, the week at which disease incidence was highest, and absolute intensity, the difference between the maximum seasonal incidence and minimum seasonal incidence [29]. The relevant curve fitted by the harmonic regression and the curve's attributes along with their interpretations are shown in Figure 2. The notations and equations for estimation can be found in Table 1. The analysis was performed for the entire period of study (July 1, 1991 through June 30, 2004) and for each influenza season; as well as for the entire population in 48 states, and for each state separately.

**Table 1:** Annual harmonic regression curve and its parameters.

Description	Notation	Expression/Commentary
**Variables of the regression model**		
Time series of disease rates	*Y*(*t*)	Per 1,000,000
Time	*t*	
Length of time series	*N*	
Length of one cycle	*M*	*M* = 52.25, for a weekly time series
**Outcomes of the regression model**		
Regression parameters for intercept, sin, and cosin components	β_0_ β_1_ β_2_	
Phase shift - distance of peak from beginning of series expressed in radians	ψ	−arctan{β_1_/β_2_}
Amplitude	*γ*	(β_1_^2^+β_2_^2^)^1/2^, if β_2_>0;
		−(β_1_^2^+β_2_^2^)^1/2^, if β_2_<0.
Standard deviations for the estimates of regression parameters β_1_ and β_2_, and covariance	σ_β1_σ_β2_σ_β1β2_	
**Curve attributes**		
Predicted seasonal curve	*S*(*t*)	
Peak Timing (expressed in weeks)Confidence Interval	*P*	*M*(1−ψ/π)/2{(β_1_σ_β1_)^2^+(β_2_σ_β2_)^2^−2β_1_β_2_σ_β1β2_}/(β_1_^2^+β_2_^2^)^2^
Absolute Intensity(Confidence Interval)	*I*	exp{β_0_+*γ*}−exp{β_0_−*γ*}exp{(β_2_σ_β1_)^2^+(β_1_σ_β2_)^2^+2β_1_β_2_σ_β1β2_}/(β_1_^2^+β_2_^2^)
Seasonal peak - maximum value	S_max_	exp{ β_0_+*γ*}
Seasonal nadir - minimum value	S_min_	exp{ β_0_−*γ*}

To obtain the national estimate of peak timing and absolute intensity we apply the model to the whole length of the time series (596 weeks) by fitting a unique curve expressed as:
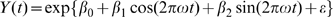
(1)where *Y*(*t*) is the weekly rate for the continental US. This model can be expanded to consider a secular trend; in our case the secular trend was negligible and the relevant terms were omitted.

Next, we estimated peak timing and absolute intensity for each of the 13 seasons by utilizing Model 2:
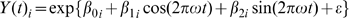
(2)where *Y*(*t*)*_i_* is weekly the rate for the continental US for *i*-season (*i* = 1–13). Number of weeks may vary in each season (52 or 53).

Finally, for each state and each season, influenza epidemics are fitted to a unique curve expressed as:
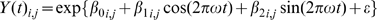
(3)where *Y*(*t*)*_i,j_* is the hospitalization rate at week *t* within a particular influenza season *i* (*i* = 1–13) for a state *j* (*j* = 1–49). In all three models, 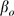 represents the intercept of the yearly epidemic curve or a baseline level; 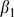 and 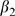 are the respective coefficients of the harmonic; and *ω* = 1/*M*, where *M* is the length of one cycle (52.25 weeks) [30]. The estimation of peak week and intensity along with their standard deviations are shown in Table 1. We also calculated the 95% confidence intervals (CI) for each of the estimates using a standard constant from a *t*-distribution of 1.96.

We utilized Spearman correlations to quantify the degree of association between peak week and absolute intensity. Using Model 2, we assessed the relationship between peak week and absolute intensity for individual seasons and Model 3 allowed us to assess this association on the state level. We also examined correlations between state centroids and seasonality characteristics.

To demonstrate a spatial pattern in peak timing we compiled the results of Model 3 as a set of 13 panels (Figure S1). For each season depicted in this set, the season is listed in the upper right-hand corner of each panel. Each panel depicts the 48 states on the y-axis in ascending order of the average peak week of the 13 influenza seasons, represented by black squares (actual values and their 95% CIs are provided in Table S1). The range of the average peak weeks across all states is shown by the highlighted orange box. Orange circles represent the current season's peak timing by state and the previous season's peak timing is shown by small, blue circles for better comparison of the differences. When all panels are shown in the proper sequence, e.g. in a chronological order of seasons, synchronization of peak timing with respect to the average can be visualized clearly, some seasons coming earlier and some later than the average.

Mapping was performed on the state level using ESRI's ArcMap GIS software (ESRI, Redlands, WA). The average peak week values highlighted in the 13 panels were categorized according to a natural breaks classification scheme with six classes.

All analysis was conducted in SAS, version 9.1 (SAS Institute) and figures were created using SPSS version 15.0.

## Results

### National Level

A 13-year weekly time series of influenza hospitalization rate per 1 million older adults has regular, well pronounced seasonal curves with the highest incidence of influenza taking place in the winter months (Figure 1). Across the 13 seasons, it appears as though the week of the highest incidence is consistently in late December to early January. The intensity varied substantially: from 358 cases in the 2002/2003 season to 7,148 cases in the 1999/2000 season at seasonal peaks. The estimates from Model 1 indicate that the average peak week was the 28.6^th^ week or the 3^rd^ week of January (95% CI: 28.44, 28.76) and that the median absolute intensity was 48.29 cases per 1 million older adults (95% CI: 45.77, 50.97).

**Figure 3:**
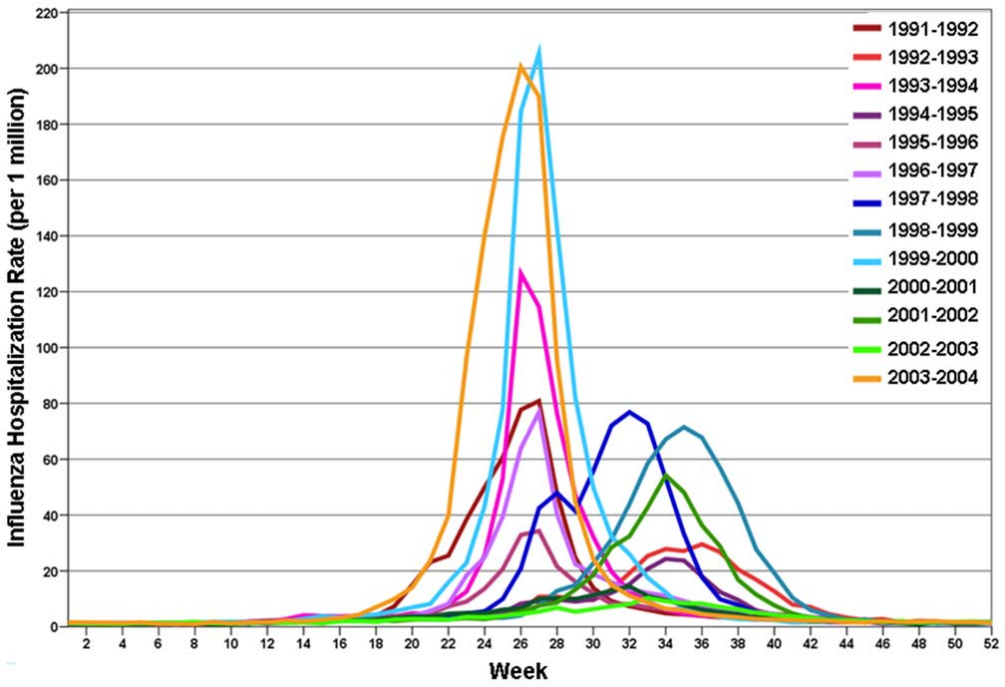
Superimposed weekly time series of influenza rate for older adults for 13 influenza seasons (1991–2004).

By superimposing a weekly time series of rates for each of the 13-seasons individually, the variability in annual weekly intensity and peak timing is evident (Figure 3). The intensities of the 1999/2000 and 2003/2004 seasons were highest. The differences in peaks become apparent: peak timing ranges from early December (week 23) during the 2003/2004 influenza season to mid-February (week 32) during the 1992/1993 influenza season. The national estimates of the peak week and intensity for each influenza season as predicted by Model 2 are shown in Table 2. In the 13 observed seasons the peak was observed within a 10-week interval: from week 23.7 in 2003/2004 to week 32.6 in 2001/2002. The predicted intensity exhibited an almost 20-fold increase from 6.3 cases per 1 million older adults in 2002/2003 to 116.1 cases per 1 million older adults in 2003/2004.

**Table 2:** National averages for peak week, absolute intensity, and their 95% confidence intervals for 13 seasons.

		MODEL 2		MODEL 3			CIRCULATING STRAINS (WHO)		
Season	Year	Peak Week (CI)	Intensity (CI)	Peak Week (CI)	Intensity (CI)	r_s_	H3N2 Strain	H1N1 Strain	B Strain
1	1991–1992	25.76 (25.30, 26.23)	42.51 (32.38, 55.81)	26.38 (25.96, 26.81)	59.47 (45.58, 73.36)	−0.46	Beijing/353/89	Singapore/6/86	Panama/45/90
2	1992–1993	33.24 (32.45, 34.05	20.30 (16.03, 25.70)	33.46 (32.93, 34.00)	30.04 (23.03, 37.05)	0.08	Beijing/353/89	Singapore/6/86	Panama/45/90
3	1993–1994	27.05 (26.66, 27.45)	51.97 (38.23, 70.65)	27.31 (26.93, 27.69)	75.15 (56.79, 93.51)	−0.36	Beijing/32/92	Singapore/6/86	Panama/45/90
4	1994–1995	32.50 (31.48, 33.53)	13.70 (10.76, 17.45)	31.99 (31.13, 32.84)	18.04 (14.12, 21.97)	0.36	Shangdong/9/93	Singapore/6/86	Panama/45/90
5	1995–1996	28.16 (27.22, 29.10)	15.57 (12.26, 19.77)	28.25 (27.64, 28.85)	23.20 (17.00, 29.41)	−0.37	Johannesburg/33/94	Singapore/6/86	Beijing/184/93
6	1996–1997	25.75 (25.16, 26.33)	31.12 (24.34, 39.78)	25.68 (25.25, 26.11)	44.25 (35.57, 52.93)	0.09	Wuhan/359/95	Singapore/6/86	Beijing/184/93
7	1997–1998	29.03 (28.62, 29.45)	50.61 (38.20, 67.06)	29.02 (28.64, 29.41)	91.62 (60.38, 122.85)	0.41	Wuhan/359/95	Bayern/7/95	Beijing/184/93
8	1998–1999	32.51 (32.08, 32.94)	48.35 (37.03, 63.14)	32.16 (31.82, 32.50)	78.27 (57.21, 99.33)	0.61	Sydney/5/97	Beijing/262/95	Beijing/184/93
9	1999–2000	26.16 (25.90, 26.42)	97.56 (69.33, 137.29)	26.19 (25.91, 26.47)	158.00 (112.07, 203.94)	−0.29	Sydney/5/97	Beijing/262/95	Beijing/184/93
10	2000–2001	29.02 (27.65, 30.39)	8.96 (6.95, 11.54)	29.23 (28.69, 29.78)	12.80 (9.76, 15.83)	0.23	Moscow/10/99	New Caldonia20/99	Beijing/184/93
11	2001–2002	32.63 (32.05, 33.22)	29.47 (22.54, 38.54)	32.31 (31.87, 32.75)	49.23 (31.86, 66.61)	0.44	Moscow/10/99	New Caldonia20/99	Sichuan/379/99
12	2002–2003	30.70 (28.88, 32.53)	6.31 (4.92, 8.08)	30.37 (29.13, 31.60)	10.67 (7.10, 14.25)	0.12	Moscow/10/99	New Caldonia20/99	Sichuan/379/99
13	2003–2004	23.74 (23.51, 23.98)	116.13 (83.46, 161.60)	23.58 (23.23, 23.93)	157.74 (129.29, 186.19)	−0.23	Moscow/10/99	New Caldonia20/99	Hong Kong/330/2001

**Figure 4:**
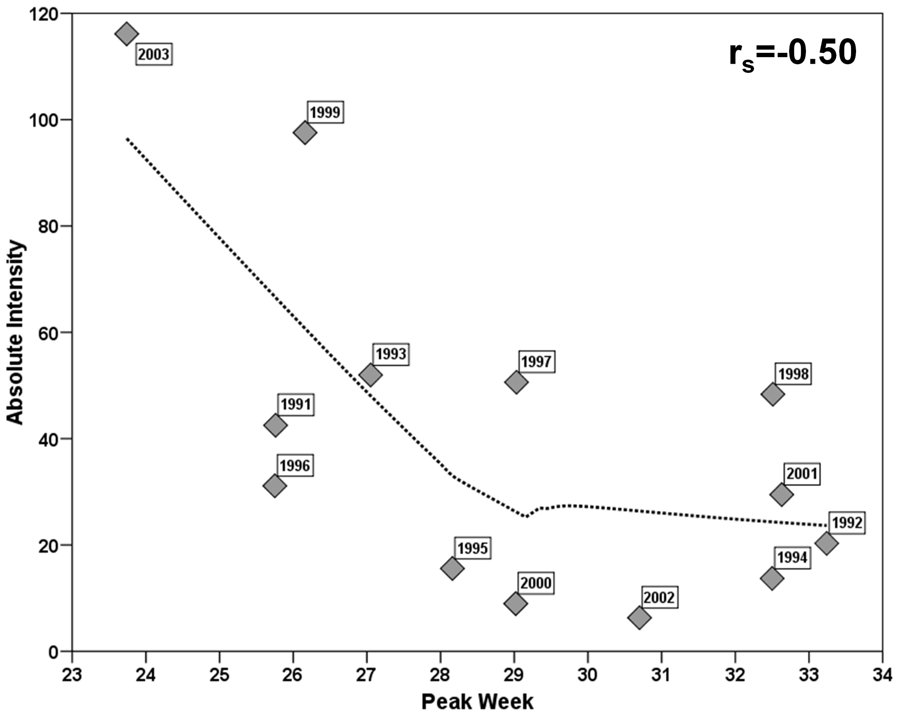
Peak week and absolute intensity for 13 influenza seasons (1991–2004).

Overall, absolute intensity and peak timing had a strong, inverse relationship (r_s_ = −0.5, p<0.05); the earlier the peak in an influenza season the more intensely the season is experienced (Figure 4).

### State Level

**Figure 5:**
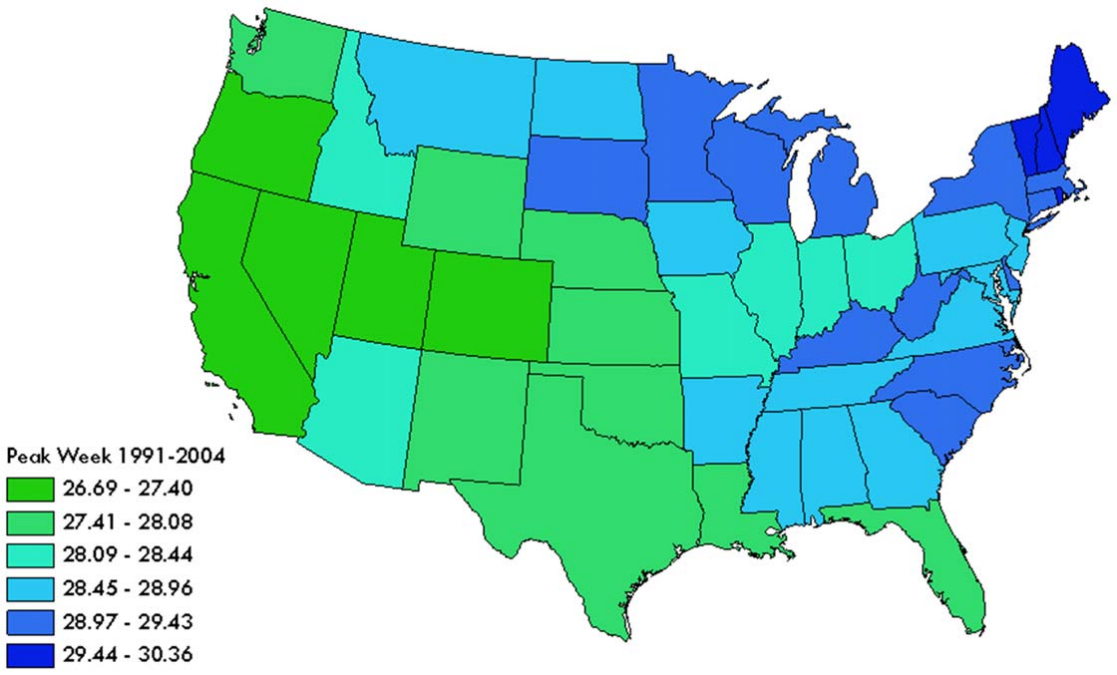
Average peak timing for 13 seasons by state derived from Model 1.

We estimated seasonality characteristics at the state levels using Model 3 (see supplemental material, Table S1). The average peak timing and intensity was estimated on a national scale by averaging the regional estimates and are shown in Table 2. Figure 5 shows the map of the average peak timing for all 13 seasons by state. Nevada, Utah, and California were the first states in terms of peak timing to experience influenza (peak weeks 26.7, 27.0, and 27.3, respectively) while, on average, Rhode Island, New Hampshire, and Maine were the last (peak weeks 29.9, 30.0, and 30.4, respectively). A west to east movement in peak week was dominant when examining the correlation between latitude and peak week (r_s_ = 0.71, p<0.01).

**Figure 6:**
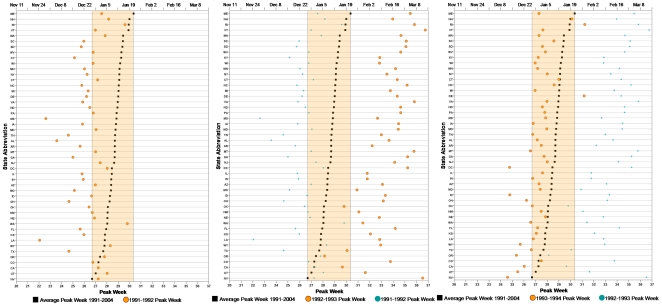
Peak week of 48 states across 13-seasons. Panel A: 1991/1992, Panel B: 1992/1993, Panel C: 1993/1994.

A dynamic movie (not included) shows the 13 seasons individually to examine synchrony with the average peak of disease. Screen shots of 3 seasons from the movie are shown in Figure 6, Panels A–C. The range of the average peak week shows that within a typical influenza season, all states will exhibit a peak within 4 weeks. Looking at synchronization with the average peak week, during the 1991/1992, 1993/1994, 1996/1997, 1999/2000, and 2003/2004 seasons, the majority of states have a peak week lower than the average peak week across all seasons while the 1992/1993, 1994/1995, 1998/1999, 2001/2002, and 2002/2003 seasons are higher than the average. The 1995/1996, 1997/1998, and 2000/2001 seasons have peak weeks which fall in line with the overall average.

**Figure 7:**
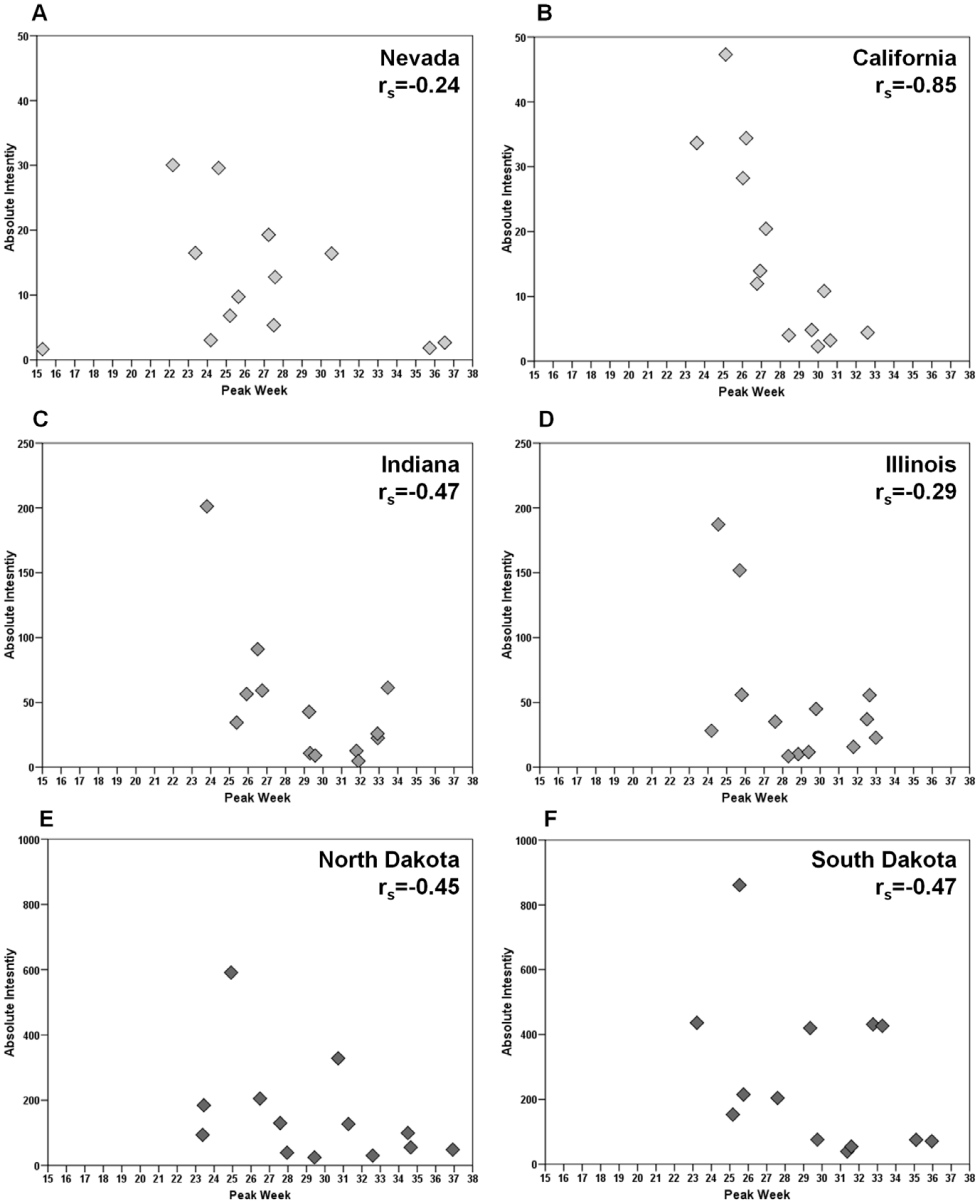
Peak week and absolute intensity for 6 selected states across 13 influenza seasons.

The estimates for intensity are highly correlated (r_s_ = 0.97) but differ substantially indicating the regional variation in hospitalization rates (Table 2, Model 3). Model 3 intensity estimates were 32%–81% higher than the estimates for Model 2. Akin to the average of seasonal peak timing, we also examined state by state variability in the relationship between peak timing and absolute intensity. Figure 7 depicts the relationship between peak week and absolute intensity for Nevada and California (Panels A and B), the two states with the lowest absolute intensity across the 13 influenza seasons; Indiana and Illinois (Panels C and D), two states with moderate absolute intensity; and North and South Dakota (Panels E and F), the two states with the highest absolute intensity. In California, a highly inverse relationship exists between the two seasonal characteristics which is not seen in a state such as Nevada (refer to Table S1 correlations).

## Discussion

We examined the national trend of influenza seasonality and the relationship between peak timing and intensity of influenza in the US older adult population and demonstrated a clear pattern in the appearance of seasonal peaks. The range of the average peak week shows that within a typical influenza season, the continental states exhibit a peak within 4 weeks with a general west to eastward spread; Nevada, Utah, and California were the first states in terms of peak timing to experience influenza while, on average, Rhode Island, New Hampshire, and Maine were the last. The early influenza seasons are typically more pronounced. These findings have a strong, practical application and are well supported by published research.

Previous research conducted to examine spatial trends of influenza demonstrated a similar spatial pattern. A study conducted by Grais, et al investigating the role of air travel in the forecasting of influenza showed similar movement patterns; researchers found a general west to east movement of influenza through the United States with some variability between influenza seasons. Focusing on the large air traffic hubs; Detroit, Los Angeles, Miami, New York City, and Philadelphia; it was demonstrated that influenza peaked first in Los Angeles in early December and ended in New York City in late February [12]. This similarity deserves special attention. In contrast to the study conducted by Grais, et al. focusing on only highly populated, urban areas, we have utilized medical records with 98% coverage for all hospitalizations in older adults residing in the continental United States. Over two-thirds of the US elderly live in rural areas, which have been shown to have disproportionately high influenza hospitalizations [34]. Therefore, it is likely that a west to east movement in influenza is a more general phenomenon than previously thought and is dictated not only by major traffic pathways but by local contact networks as well.

We found that individual influenza seasons show synchrony with consistent late or early timing occurring across all 48 states during each influenza season in comparison to the average across the 13 influenza seasons. In a study by Brownstein et al. measuring the rate of inter-regional spread and timing of influenza in the United States for nine influenza seasons, researchers found that influenza took 2 weeks to peak over all United States regions. Using nine broad United States Census Bureau defined regions, the researchers observed similarities between the spread of influenza between seasons; specifically, geographical patterns were synchronized within individual seasons, similar to findings presented in this report. Variation was witnessed only in the week at which influenza peaked between each season. However, the authors commented that limited data available for subgroup analysis, such as by age group or on a regional level, may be overlooking detailed patterns in the spread and timing of influenza [13], which our study accomplished through analysis of older adults on the state level. It is important to distinguish the range of seasonal peaks observed from year to year from regional peaks for a given influenza season. Our study showed that in 13 observed seasons there was a 10 week interval in which an overall peak can be observed, however for a given season, on average, all states experienced a peak with 4 weeks.

In the present study, we aimed to examine the relationship between peak timing and intensity of influenza seasons in order to establish a base for predicting influenza. We found that across all 13 influenza seasons, peak timing and absolute intensity were significantly, inversely related; earlier seasons are more likely to have more cases of influenza on both the regional and national levels. While the inverse correlation is primarily driven by the 1999–2000 and 2003–2004 influenza seasons, it is through these outlying seasons that important public health implications can be made. For example, the 2003–2004 influenza season experienced higher than usual intensity due to a vaccine mismatch. The higher than average severity in 1999 has been hypothesized to be linked to the particularly severe 1997–1998 influenza seasons in the swine population [35]. Analyzing pneumonia and influenza deaths from 1972–1997, Viboud et al. examined the synchrony and timing of influenza epidemics in the United States as well as in France and Australia. The authors found a high level of synchrony in the peak timing and amplitude of influenza in the United States and France where earlier influenza seasons showed higher mortality rates from pneumonia and influenza both within and between France and the United States [22]. These observations held true for their assessment of synchrony on a state level [15]. Our findings show that peak timing and absolute intensity exhibited such relationship not only for mortality but for morbidity as well. This is important for influenza-related hospitalizations that typically occur due to complications, so virological causes of lower-respiratory tract infections in older adults are rarely investigated in detail and are sensitive to the decision to test for diagnosis by the clinician [36]. From the point of view of prevention, our findings demonstrate the value of two complimentary parameters that can be adapted for influenza surveillance: the timing of influenza and absolute intensity to serve as a proxy for the time period we can expect the highest incidence of disease and the severity of infection.

In the present study, we estimated peak timing using the δ-method [30]–[31] applied to parameters of harmonic regression models, also known as Serfling regression of counts [7]. Serfling-type periodic regression models have been widely used to establish the (unobserved) seasonal baseline of influenza [6]–[7], [22], [33]. Estimation of seasonal baseline is a challenging statistical problem because the observations comprising baseline versus epidemic activity are not identifiable. The estimates of weekly incidence from these models can be potentially biased if the influence of epidemics is not taken into account. The estimates of peak timing presented in this study most likely reflect the most dominant peak in a mixture of peaks that occurred in a given location during an influenza season. Due to the robustness of the hospitalization data utilized for our analysis, it is highly unlikely that the estimates of the peak week will be driven by only a few observations. Unless there is a substantial deviation from the sharp spikes observed seasonally, which were not witnessed as seen in Figure 1, the δ-method provides stabile estimates of variation in the characteristics of a seasonal pattern. Our intent was to estimate the point in time when an outcome of interest, e.g. hospitalization rate, reaches its maximum intensity without separating specific outbreaks from an unobservable baseline. The alternative approach might be to record the peak week as the modal observation of the seasonal time series, however, data-driven, non-parametric ranking is associated with a number of serious flaws. With data on well-documented outcomes, e.g. strain-specific, laboratory-confirmed counts, the proposed approach would allow to estimate peak time directly from the fitted curve and to systematically and comprehensively compare multiple outbreaks with a high degree of precision. Furthermore, further analysis using the proposed fit to a short time series that includes a single season or a number of seasons, e.g. with similar circulation strains or vaccination coverage, will facilitate a focused comparison of seasonal dynamics.

Incomplete testing for influenza in hospitalization records and the lack of control for circulating influenza strains in our models limit our inference to causal pathogenicity, although we have made an attempt to consider the role of strain in Figure S1. Previous research has shown an association between circulating H3N2 and B strains in the second season of their circulation and changes in intensity and peak timing of disease [15]–[17]. One of the difficulties of incorporating this information into the models was the inaccuracy of circulating influenza strain reporting on a regional level. Circulating strain can be closely approximated by the active strains used for vaccination as recommended by the World Health Organization (WHO). However, reports by the Centers for Disease Control and Prevention (CDC) on circulating strains based on confirmatory testing do not always match the circulating strains reported by WHO. We made an attempt to describe these discrepancies (see Table 3). For example, during the epidemic 1999–2000 influenza season, the H1N1 component of the vaccine contained Beijing/262/95. However, only 1% of the H1N1 strains tested nationally matched the Beijing strain. Sixty-seven percent of the strains tested matched New Caladonia/20/99 [37]. Furthermore, during the 2001–2002 season, neither the H3N2 nor the B strains in the vaccine matched the predominant circulating strains reported by the CDC. Reports on circulating strains issued by the CDC rarely report geographic variability which is likely a contributor to the variability seen both on a seasonal and state-by-state level in the current analysis [38].

**Table 3:** WHO versus CDC circulating influenza strains (H3N2, H1N1, and B) for 13 seasons.

Season	H3N2 Strain (WHO)	H3N2 Strain (CDC)**	H1N1 Strain (WHO)	H1N1 Strain (CDC)	B Strain (WHO)	B Strain (CDC)
1991–1992	Beijing/353/89*	Beijing/353/89*	Singapore/6/86	Taiwan/01/86	Panama/45/90*	Panama/45/90*
1992–1993	Beijing/353/89	Beijing/32/92Shangdong/9/93	Singapore/6/86	Taiwan/01/86Texas/36/91	Panama/45/90*	Panama/45/90*
1993–1994	Beijing/32/92*	Beijing/32/92*	Singapore/6/86	Taiwan/01/86Texas/36/91	Panama/45/90*	Panama/45/90*
1994–1995	Shangdong/9/93*	Shangdong/9/93*Johannesburg/33/94	Singapore/6/86	Taiwan/01/86 (50%)Texas/36/91 (50%)	Panama/45/90*	Panama/45/90*Beijing/184/93Harbin/7/94Shanghai/04/94
1995–1996	Johannesburg/33/94*	Johannesburg/33/94*Wuhan/359/95	Singapore/6/86	Taiwan/01/86 (50%)Texas/36/91 (50%)	Beijing/184/93*	Beijing/184/93*Harbin/7/94
1996–1997	Wuhan/359/95*	Wuhan/359/95*Nanchang/933/95	Singapore/6/86	Bayern/7/95	Beijing/184/93*	Beijing/184/93*Harbin/7/94
1997–1998	Wuhan/359/95*	Wuhan/359/95* (19%)Sydney/5/97 (81%)	Bayern/7/95*	Bayern/7/95* (43%)Johannesburg/82/96 (43%)Beijing/262/95 (14%)	Beijing/184/93*	Beijing/184/93*Harbin/7/94
1998–1999	Sydney/5/97*	Sydney/5/97* (90%)	Beijing/262/95*	Beijing/262/95*Bayern/7/95	Beijing/184/93*	Beijing/184/93* (100%)
1999–2000	Sydney/5/97*	Sydney/5/97* (94%)	Beijing/262/95*	Beijing/262/95* (1%)New Caladonia/20/99 (67%)Bayern/7/95 (32%)	Beijing/184/93*	Beijing/184/93* (100%)
2000–2001	Moscow/10/99	Panama/2007/99	New Caladonia/20/99*	New Caladonia/20/99*	Beijing/184/93	Hong Kong/330/2001
2001–2002	Moscow/10/99	Panama/2007/99 (100%)	New Caladonia/20/99*	New Caladonia/20/99* (100%)	Sichuan/379/99	Yamagata/16/88 (23%)*-Similar to Sichuan*Victoria/2/87 (77%)
2002–2003	Moscow/10/99	Panama/2007/99 (93%)	New Caladonia/20/99*	New Caladonia/20/99* (100%)	Sichuan/379/99	Hong Kong/330/2001 (99%)Yamagata/16/88 (1%)*-Similar to Sichuan*
2003–2004	Moscow/10/99	Panama/2007/99 (11%)Fujian/411/2002 (89%)	New Caladonia/20/99*	New Caladonia/20/99* (100%)	Hong Kong/330/2001	Yamagata/16/88 (93%)Victoria/2/87 (7%)*-Similar to Hong Kong*

*Matching WHO and CDC strains for each of the corresponding seasons and influenza strains.

**Percentages (in parentheses) represent the specimens tested positive for each of the circulating strains.

Yearly parameters captured by the proposed approach allow for analysis of complex, non-linear trends over a long time frame, examination of characteristics of individual seasons, and assessment of inter-season heterogeneity and intra-season correlations. Using complex modeling techniques, researchers can determine vaccination practices for vulnerable populations and state level clinical interventions can be enhanced by forecasting the expected intensity of disease and the timing of the disease's peak in specific subpopulations and geographical locations. Understanding the geographical patterns of influenza spread and utilizing multiple parameters for predictive modeling are essential for guiding prevention efforts.

## Supporting Information

Figure S1 Compiled the results of Model 3 as a set of 13 panels. Each panel depicts the 48 states in ascending order of the average peak week of the 13 influenza seasons.
(1.37 MB DOC)

Table S1 Seasonality characteristics at the state level using Model 3.
(0.29 MB DOC)
