# Supplementary material for: Seasonal Synchronization of Influenza in the United States Older Adult Population
Source: PLoS One. 2010 Apr 15;5(4):e10187. doi: 10.1371/journal.pone.0010187 (PMC2855366; doi:10.1371/journal.pone.0010187)
Supplement: Figure S1 — Compiled the results of Model 3 as a set of 13 panels. Each panel depicts the 48 states in ascending order of the average peak week of the 13 influenza seasons. (1.37 MB DOC) [file pone.0010187.s001.doc]

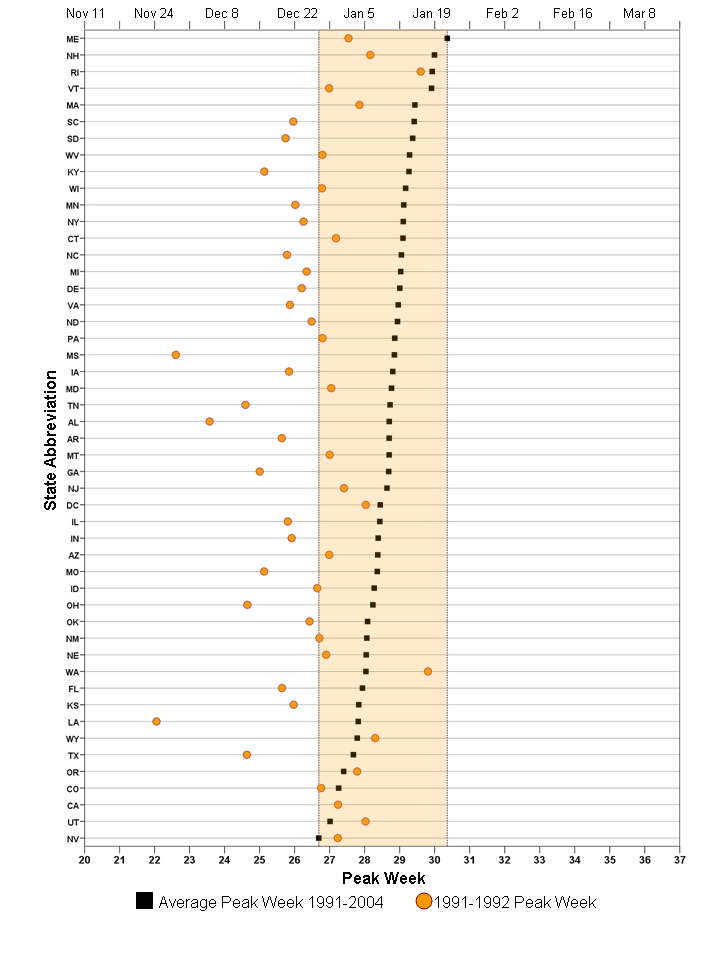


Figure S1, Panel A: 1991-1992 Influenza Season <Beijing/353/89, Singapore/6/86, Panama/45/90>


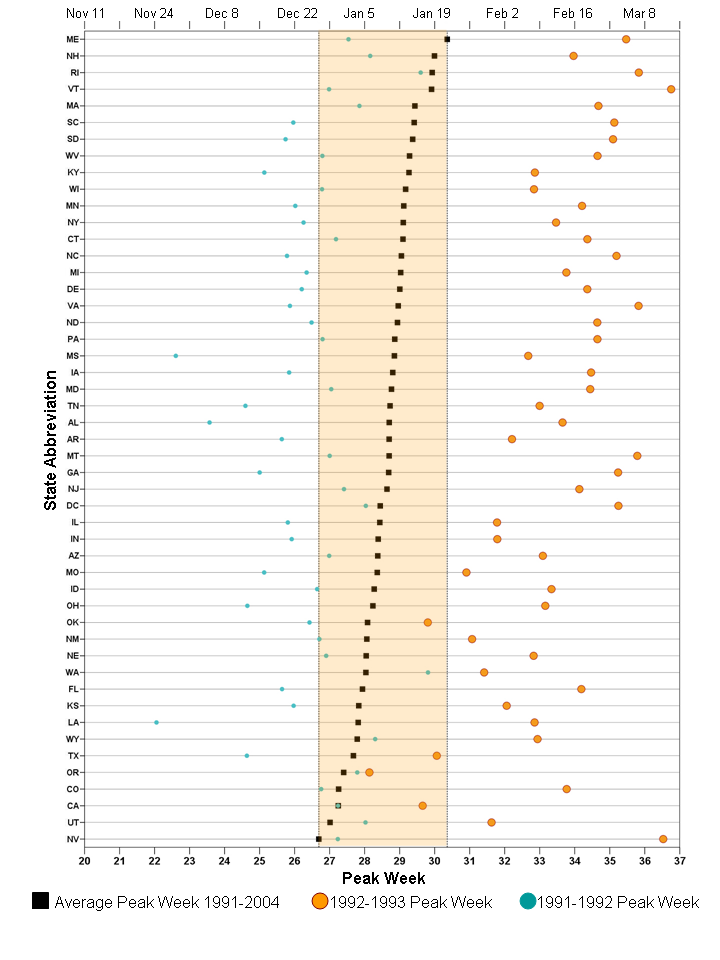


Figure S1, Panel B: 1992-1993 Influenza Season <Beijing/353/89, Singapore/6/86, Panama/45/90>


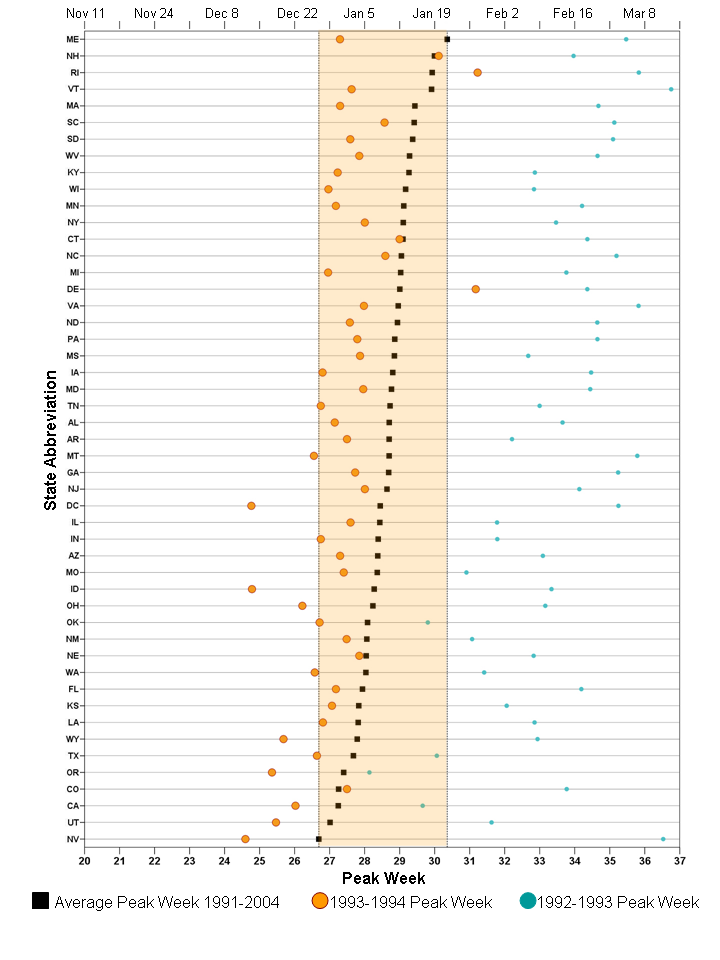


Figure S1, Panel C: 1993-1994 Influenza Season <Beijing/32/92, Singapore/6/86, Panama/45/90>


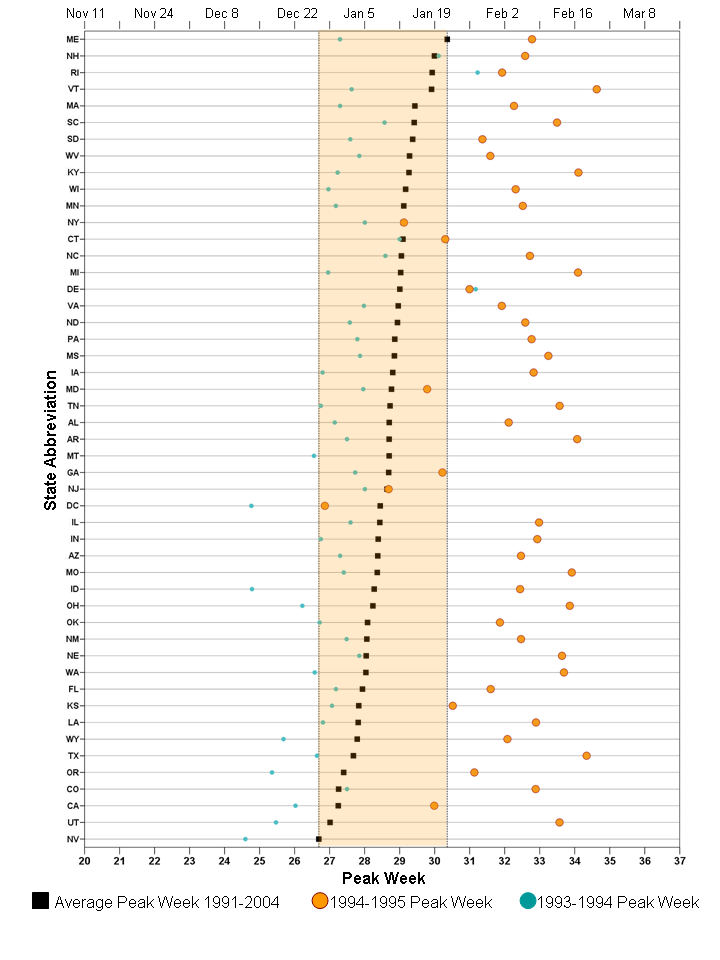


Figure S1, Panel D: 1994-1995 Influenza Season <Shangdong/9/93, Singapore/6/86, Panama/45/90


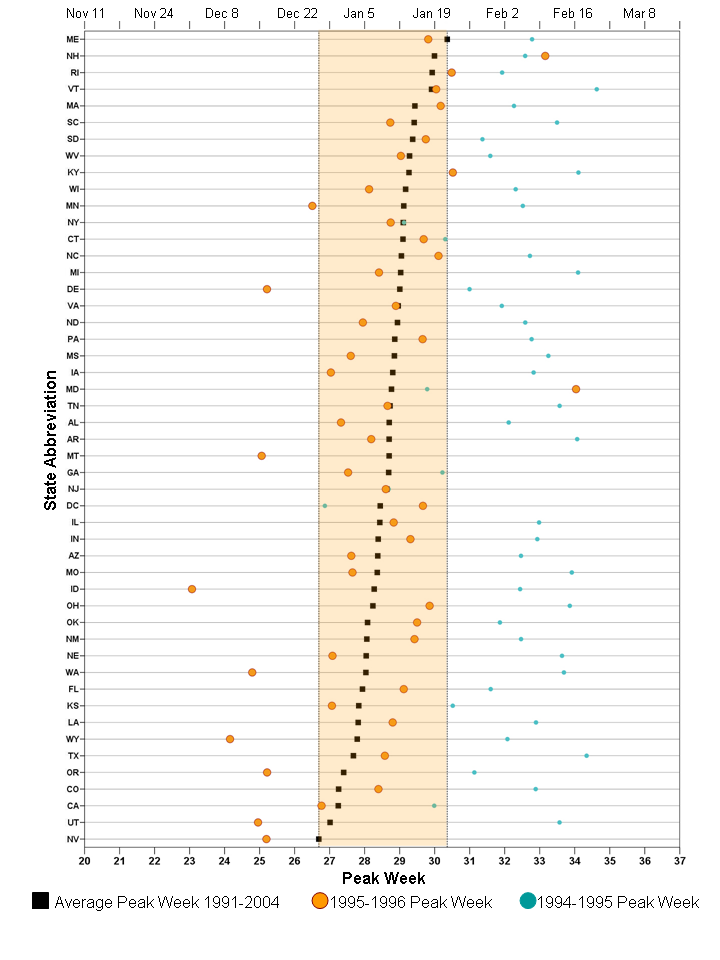


Figure S1, Panel E: 1995-1996 Influenza Season <Johannesburg/33/94, Singapore/6/86, Beijing/184/93>


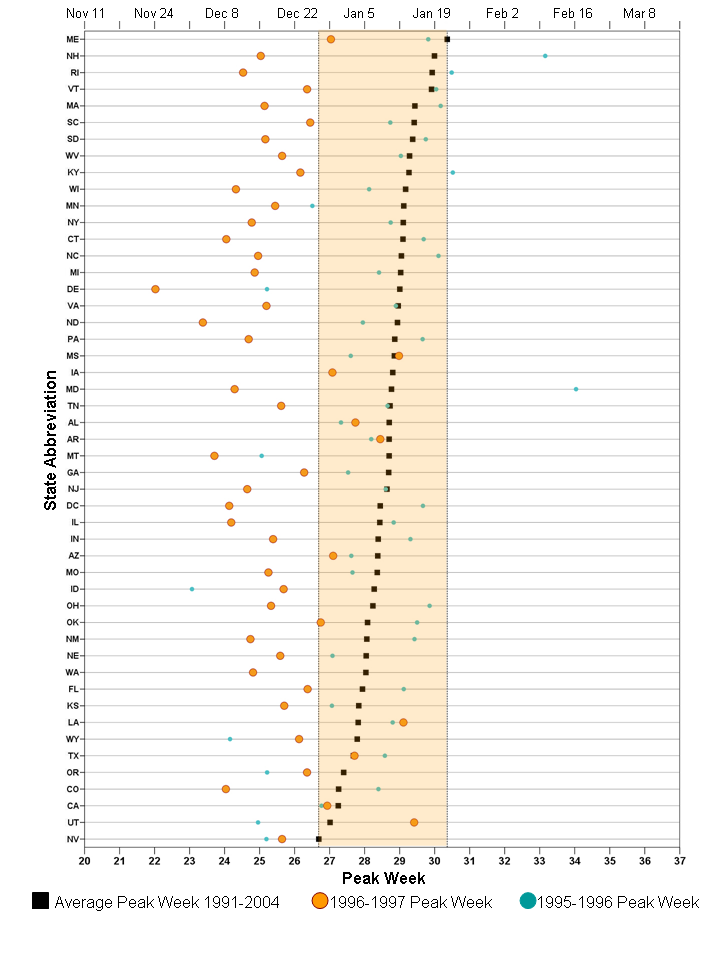


Figure S1, Panel F: 1996-1997 Influenza Season <Wuhan/359/95, Singapore/6/86, Beijing/184/93>


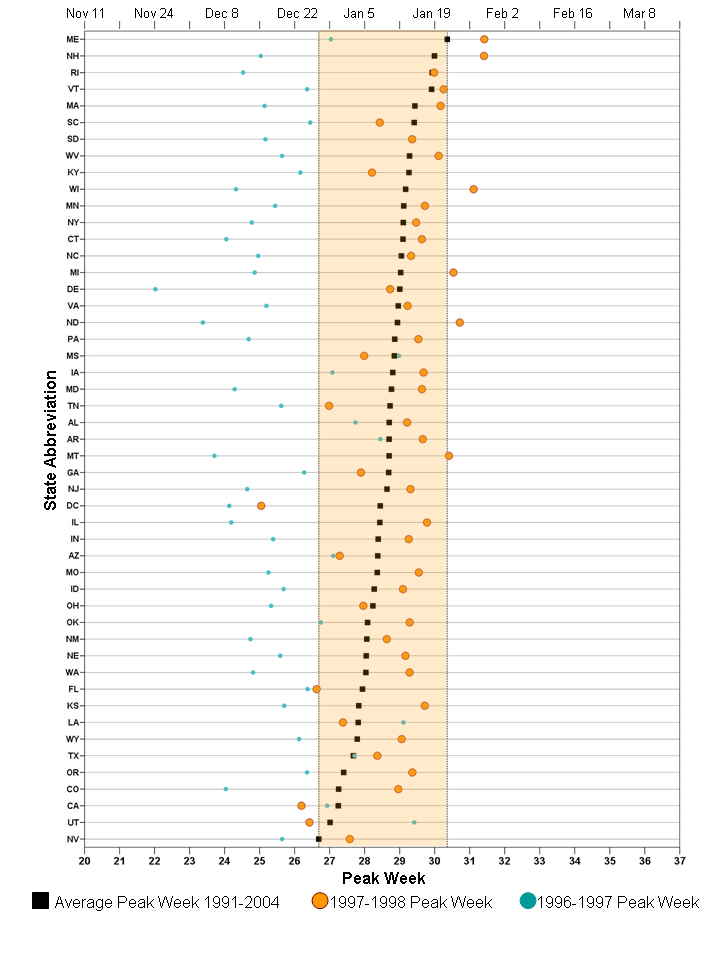


Figure S1, Panel G: Peak 1997-1998 Influenza Season <Wuhan/359/95, Bayern/7/95, Beijing/184/93>


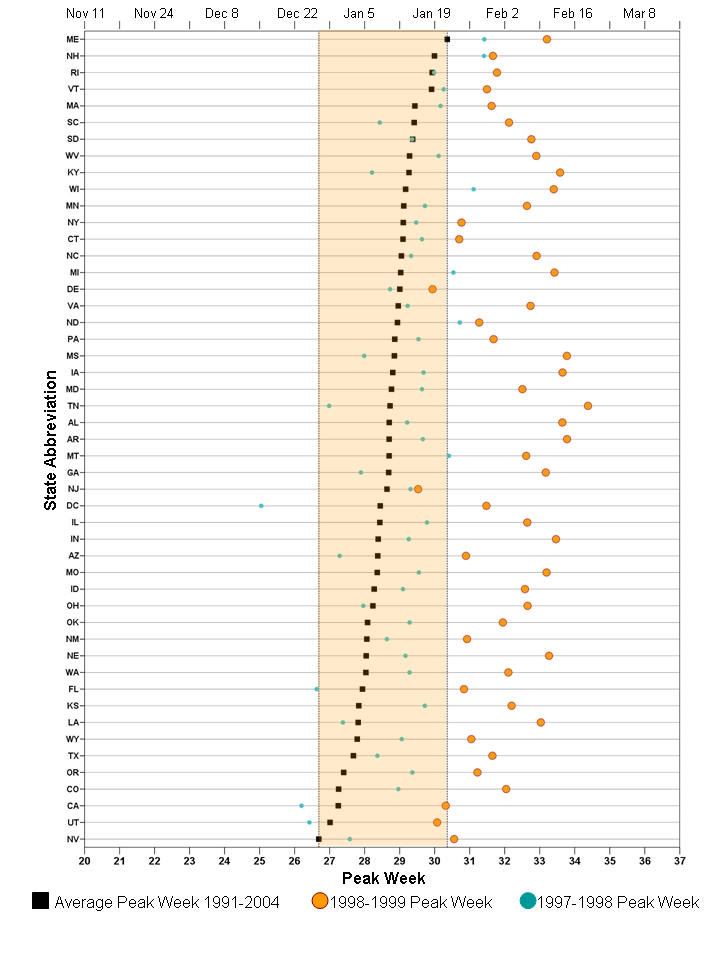


Figure S1, Panel H: 1998-1999 Influenza Season <Sydney/5/97, Beijing/262/95, Beijing/184/93>


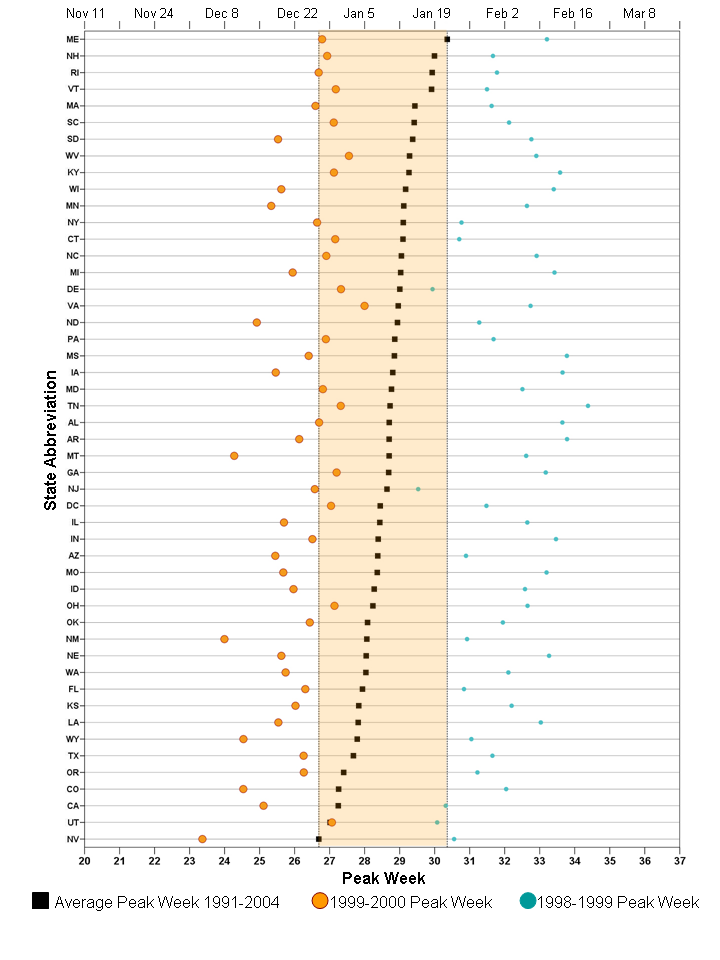


Figure S1, Panel I: 1999-2000 Influenza Season <Sydney/5/97, Beijing/262/95, Beijing/184/93>


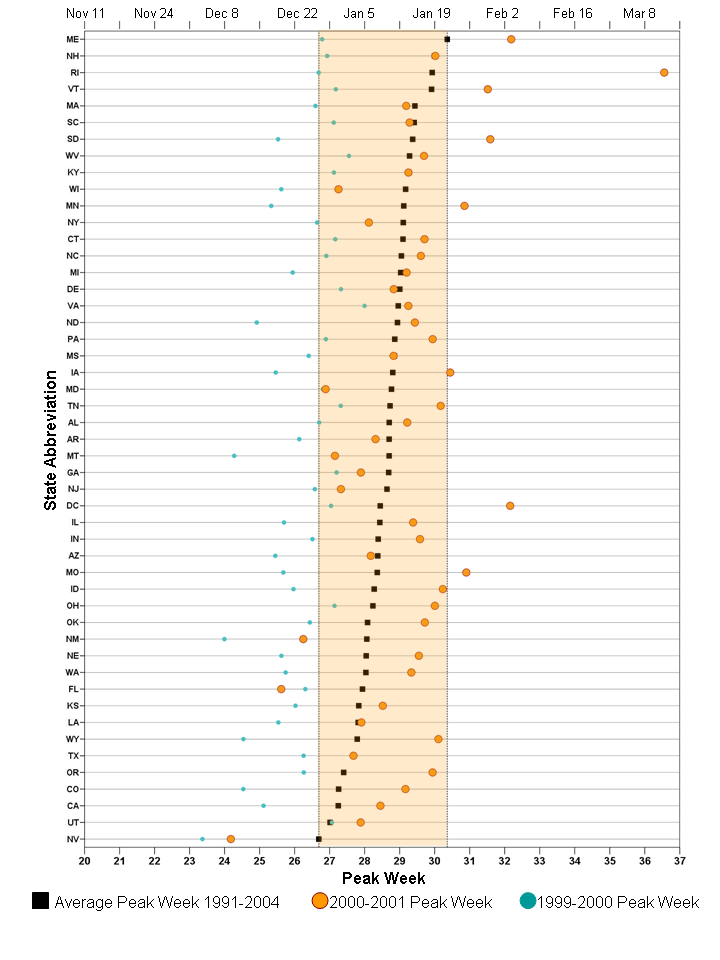


Figure S1, Panel J: 2000-2001 Influenza Season <Moscow/10/99, New Caldonia20/99, Beijing/184/93>


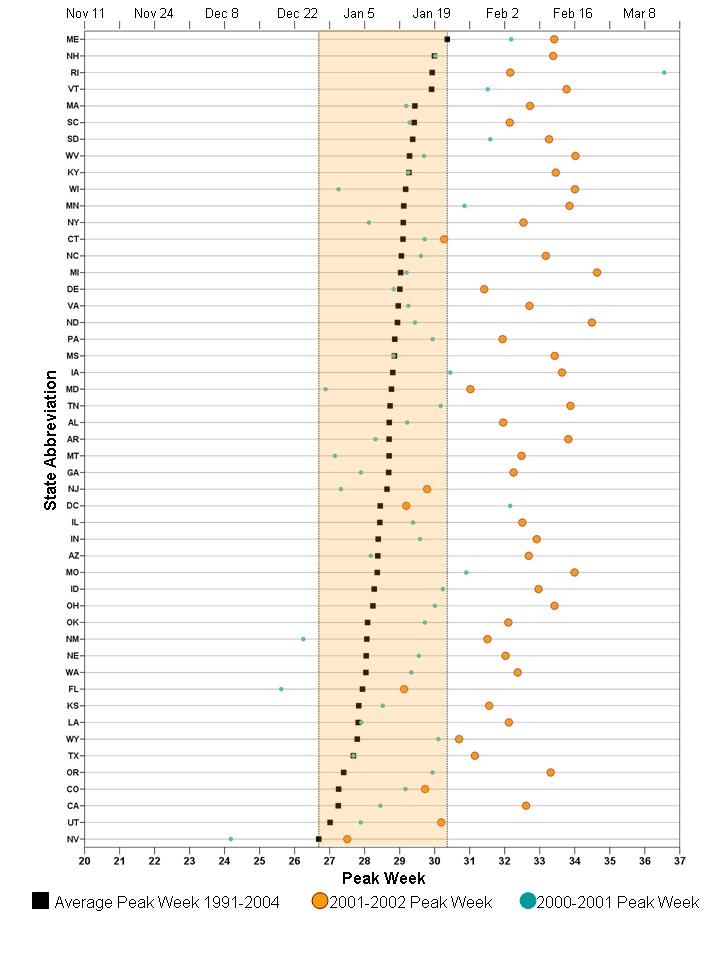


Figure S1, Panel K: 2001-2002 Influenza Season <Moscow/10/99, New Caldonia20/99, Sichuan/379/99>


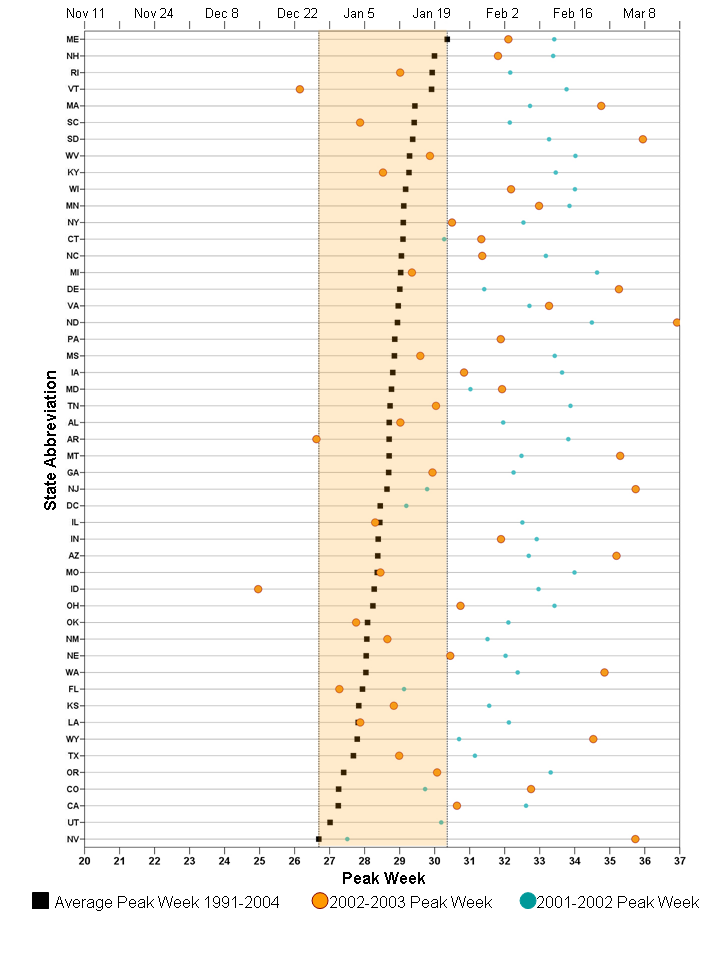


Figure S1, Panel L: 2002-2003 Influenza Season <Moscow/10/99, New Caldonia20/99, Sichuan/379/99>


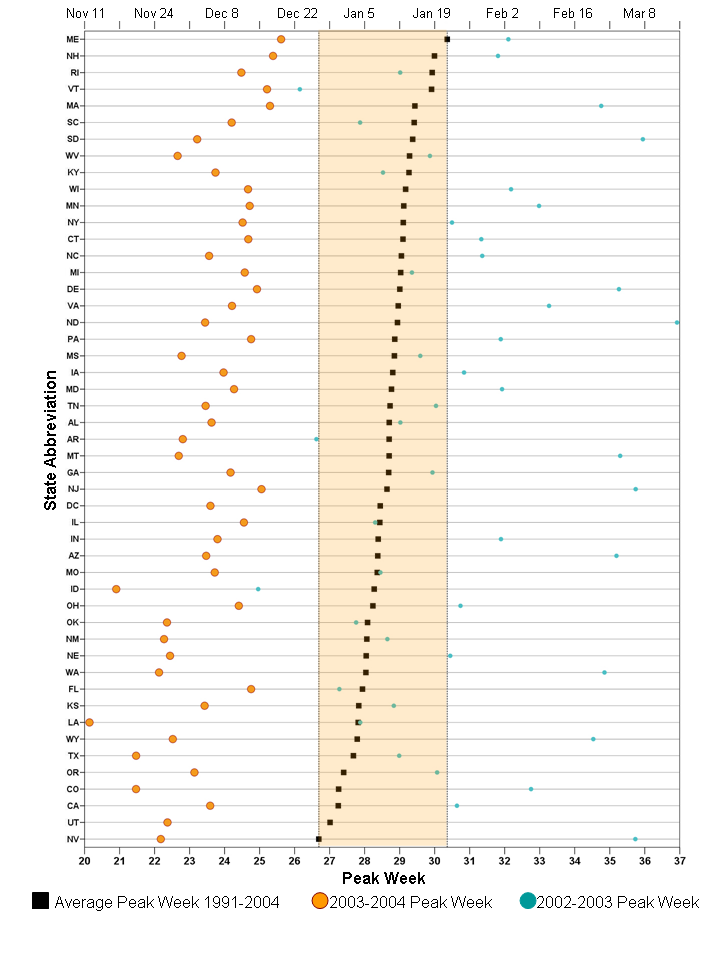


Figure S1, Panel M: 2003-2004 Influenza Season <Moscow/10/99, New Caldonia20/99, Hong Kong/330/2001>
