# Supplementary material for: Seasonal Synchronization of Influenza in the United States Older Adult Population
Source: PLoS One. 2010 Apr 15;5(4):e10187. doi: 10.1371/journal.pone.0010187 (PMC2855366; doi:10.1371/journal.pone.0010187)
Supplement: Table S1 — Seasonality characteristics at the state level using Model 3. (0.29 MB DOC) [file pone.0010187.s002.doc]

| **State** | **Overall** | **Correlation*** | **1991-92** | **1992-93** | **1993-94** | **1994-95** | **1995-96** | **1996-97** | **1997-98** | **1998-99** | **1999-00** | **2000-01** | **2001-02** | **2002-03** | **2003-04** |
| --- | --- | --- | --- | --- | --- | --- | --- | --- | --- | --- | --- | --- | --- | --- | --- |
| Nevada | 26.69  (26.38, 27.00)  9.62  (8.74, 10.59) | -0.24 | 27.23  (26.62, 27.85)  19.29  (10.20, 36.47) | 36.53  (33.57, 39.49)  2.66  (1.87, 3.78) | 24.59  (24.13, 25.06)  29.60  (14.27, 61.40) | 15.30  (10.77, 19.83)  1.65  (1.17, 2.34) | 25.19  (23.73, 26.66)  6.81  (4.87, 9.54) | 25.64  (24.43, 26.89)  9.74  (7.29, 13.00) | 27.57  (26.79, 28.36)  12.77  (6.51, 25.06) | 30.56  (29.83, 31.28)  16.41  (10.08, 26.70) | 23.37  (22.61, 24.12)  16.49  (11.00, 24.77) | 24.18  (21.84, 26.52)  3.04  (1.96, 4.72) | 27.50  (25.79, 29.22)  5.35  (3.76, 7.61) | 35.73  (31.54, 39.92)  1.84  (1.31, 2.58) | 22.18  (21.67, 22.68)  30.05  (18.90, 47.80) |
| Utah | 27.01  (26.79, 27.23)  21.47  (20.29, 22.71) | -0.34 | 28.02  (27.10, 28.95)  13.52  (9.76, 18.73) | 31.62  (30.28, 32.97)  8.51  (6.39, 11.31) | 25.47  (25.05, 25.88)  38.73  (21.98, 68.24) | 33.47  (30.30, 36.83)  2.37  (1.67, 3.37) | 24.95  (24.09, 25.81)  19.83  (16.28, 24.14) | 29.41  (27.85, 30.98)  11.52  (9.93, 13.35) | 26.42  (25.58, 27.27)  19.14  (15.40, 23.78) | 30.07  (29.18, 30.96)  22.23  (19.04, 25.95) | 27.06  (26.52, 27.60)  42.25  (35.59, 50.15) | 27.89  (25.75, 30.02)  6.14  (5.01, 7.52) | 30.19  (29.30, 31.07)  18.19  (14.71, 22.49) | 15.44  (9.55, 21.33)  1.85  (1.47, 2.32) | 22.37  (22.19, 22.55)  160.43  (97.13, 264.99) |
| California | 27.25  (27.01, 27.49)  14.98  (13.71, 16.36) | -0.85 | 27.24  (26.51, 27.97)  20.43  (15.37, 27.16) | 29.66  (27.54, 31.77)  4.82  (3.65, 6.36) | 26.02  (25.47, 26.58)  28.24  (19.49, 40.93) | 29.99  (26.24, 33.73)  2.29  (1.69, 3.11) | 26.77  (25.75, 27.78)  11.97  (8.72, 16.42) | 26.93  (26.01, 27.85)  13.94  (10.23, 19.00) | 26.19  (25.71, 26.68)  34.40  (23.04, 51.37) | 30.31  (29.21, 31.41)  10.82  (7.97, 14.69) | 25.11  (24.74, 25.48)  47.31  (28.06, 79.74) | 28.45  (26.37, 30.53)  4.00  (2.77, 5.79) | 32.61  (30.52, 34.70)  4.42  (3.21, 6.07) | 30.64  (27.99, 33.28)  3.21  (2.31, 4.47) | 23.59  (23.11, 24.06)  33.66  (21.40, 52.97) |
| Colorado | 27.26  (27.10, 27.41)  33.21  (30.87, 35.73) | -0.53 | 26.76  (26.28, 27.23)  34.45  (22.69, 52.30) | 33.77  (32.48, 35.06)  8.60  (6.34, 11.67) | 27.50  (26.80, 28.19)  19.57  (13.49, 28.39) | 32.88  (32.12, 33.65)  15.57  (9.95, 24.37) | 28.39  (27.31, 29.48)  11.59  (8.75, 15.34) | 24.03  (23.43, 24.64)  28.60  (22.10, 37.00) | 28.96  (28.60, 29.33)  45.31  (23.53, 87.24) | 32.04  (31.73, 32.36)  53.32  (23.53, 120.81) | 24.53  (24.28, 24.79)  87.82  (50.65, 152.27) | 29.17  (28.21, 30.12)  12.19  (8.46, 17.56) | 29.73  (29.31, 30.14)  50.19  (37.46, 67.25) | 32.75  (31.83, 33.67)  14.15  (10.50, 19.07) | 21.47  (21.29, 21.65)  193.32  (141.54, 264.04) |
| Oregon | 27.40  (27.22, 27.58)  22.51  (20.59, 24.62) | -0.64 | 27.79  (27.12, 28.45)  23.67  (17.77, 31.51) | 28.13  (27.43, 28.84)  21.25  (15.83, 28.52) | 25.35  (25.10, 25.60)  96.85  (62.90, 149.13) | 31.13  (28.55, 33.72)  3.29  (2.36, 4.58) | 25.21  (24.35, 26.07)  14.44  (10.14, 20.56) | 26.35  (25.92, 26.79)  41.53  (28.85, 59.78) | 29.36  (28.44, 30.28)  14.65  (11.06, 19.39) | 31.22  (30.62, 31.82)  23.91  (15.83, 36.10) | 26.26  (25.75, 26.77)  31.83  (21.46, 47.22) | 29.94  (28.20, 31.68)  5.14  (3.58, 7.37) | 33.31  (32.35, 34.28)  10.90  (6.99, 16.99) | 30.07  (27.09, 33.05)  2.23  (1.44, 3.56) | 23.14  (22.79, 23.48)  53.12  (30.94, 91.21) |
| Texas | 27.68  (27.52, 27.84)  33.53  (31.64, 35.54) | -0.57 | 24.64  (24.12, 25.15)  34.13  (25.23, 46.16) | 30.06  (29.20, 30.92)  18.22  (14.52, 22.86) | 26.63  (26.36, 26.91)  92.00  (64.39, 131.45) | 34.34  (33.02, 35.66)  10.48  (8.43, 13.04) | 28.58  (27.80, 29.35)  22.33  (18.21, 27.39) | 27.71  (27.09, 28.33)  29.85  (24.02, 37.11) | 28.36  (27.79, 28.93)  34.07  (27.47, 42.26) | 31.65  (31.15, 32.15)  39.02  (30.26, 50.31) | 26.26  (25.98, 26.53)  90.92  (63.99, 129.21) | 27.68  (26.34, 29.02)  9.83  (7.81, 12.38) | 31.15  (30.54, 31.76)  29.71  (23.45, 37.64) | 28.99  (27.12, 30.85)  6.75  (5.42, 8.40) | 21.47  (21.18, 21.76)  98.73  (79.21, 123.06) |
| Wyoming | 27.79  (27.69, 27.88)  81.81  (78.29, 85.48) | -0.67 | 28.30  (27.94, 28.66)  87.06  (76.57, 99.00) | 32.94  (32.01, 33.87)  28.94  (26.15, 32.03) | 25.68  (25.48, 25.88)  162.68  (122.90, 215.33) | 32.08  (31.37, 32.79)  25.32  (20.58, 31.14) | 24.15  (23.61, 24.70)  33.43  (25.81, 43.30) | 26.13  (25.45, 26.80)  34.75  (30.28, 39.89) | 29.06  (28.86, 29.26)  170.59  (132.71, 219.28) | 31.05  (30.84, 31.26)  139.85  (97.13, 201.34) | 24.54  (24.40, 24.67)  306.40  (225.61, 416.14) | 30.11  (29.39, 30.82)  26.20  (21.62, 31.74) | 30.70  (30.35, 31.04)  70.08  (53.53, 91.74) | 34.53  (33.89, 25.17)  24.31  (17.74, 33.29) | 22.52  (22.36, 22.68)  258.53  (207.77, 321.69) |
| Louisiana | 27.81  (27.69, 27.94)  52.97  (50.43, 55.64) | -0.62 | 22.05  (21.76, 22.36)  87.61  (68.63, 111.82) | 32.85  (32.44, 33.26)  57.05  (45.93, 70.87) | 26.81  (26.56, 27.05)  125.36  (97.72, 160.82) | 32.89  (32.15, 33.64)  23.89  (19.49, 29.27) | 28.80  (28.45, 29.15)  64.76  (48.87, 85.82) | 29.11  (28.63, 29.59)  45.90  (37.54, 56.13) | 27.38  (27.06, 27.70)  86.26  (70.45, 105.62) | 33.03  (32.57, 33.49)  45.48  (35.85, 57.69) | 25.53  (25.36, 25.70)  192.99  (130.71, 284.95) | 27.91  (27.16, 28.66)  21.81  (17.24, 27.59) | 32.12  (31.51, 32.72)  31.87  (25.88, 39.25) | 27.87  (26.65, 29.09)  12.24  (10.01, 14.97) | 20.14  (19.76, 20.52)  68.42  (57.12, 81.94) |
| Kansas | 27.83  (27.74, 27.93)  77.92  (73.97, 82.08) | -0.47 | 25.97  (25.66, 26.28)  90.27  (73.78, 110.44) | 32.06  (31.51, 32.60)  41.84  (35.29, 49.61) | 27.07  (26.87, 27.26)  166.99  (121.89, 228.79) | 30.52  (29.38, 31.65)  14.78  (12.39, 17.63) | 27.06  (26.32, 27.81)  25.61  (21.38, 30.68) | 25.70  (25.38, 26.03)  77.17  (59.45, 100.17) | 29.72  (29.38, 30.05)  78.95  (63.92, 97.52) | 32.20  (31.89, 32.51)  78.46  (58.76, 104.77) | 26.02  (25.86, 26.19)  220.35  (166.39, 291.79) | 28.52  (27.55, 29.49)  18.66  (15.77, 22.07) | 31.56  (31.14, 31.97)  55.42  (44.81, 68.54) | 28.83  (27.74, 29.93)  14.12  (11.55, 17.27) | 23.43  (23.30, 23.56)  345.63  (266.92, 447.54) |
| Florida | 27.94  (27.63, 28.24)  11.27  (10.40, 12.20) | -0.45 | 25.64  (24.70, 28.58)  12.83  (9.16, 17.99) | 34.19  (32.32, 36.06)  5.20  (3.82, 7.08) | 27.18  (26.52, 27.83)  21.36  (14.58, 31.30) | 31.60  (29.56, 33.64)  4.36  (3.11, 6.12) | 29.12  (27.46, 30.77)  6.09  (4.47, 8.30) | 26.37  (24.76, 27.98)  6.91  (5.26, 9.06) | 26.63  (25.48, 27.77)  10.38  (7.71, 13.98) | 30.84  (29.60, 32.07)  10.07  (7.75, 13.07) | 26.30  (25.68, 26.92)  27.36  (21.01, 35.62) | 25.62  (23.05, 28.19)  3.56  (2.63, 4.82) | 29.12  (27.63, 30.62)  7.61  (5.80, 10.00) | 27.28  (22.91, 31.64)  2.04  (1.53, 2.72) | 24.76  (24.32, 25.19)  42.63  (29.73, 61.11) |
| Washington | 28.03  (27.85, 28.22)  23.81  (22.08, 25.67) | -0.62 | 29.81  (28.82, 30.80)  13.50  (10.32, 17.66) | 31.41  (30.69, 32.14)  20.85  (15.67, 27.74) | 26.57  (26.25, 26.90)  68.89  (48.52, 97.80) | 33.69  (32.00, 35.39)  6.15  (4.59, 8.23) | 24.79  (23.70, 25.87)  11.27  (8.41, 15.11) | 24.81  (24.34, 25.29)  38.27  (27.78, 52.71) | 29.28  (28.63, 29.93)  23.80  (17.53, 32.32) | 32.10  (31.63, 32.58)  36.47  (25.29, 52.60) | 25.74  (25.41, 26.08)  59.59  (38.33, 92.65) | 29.33  (27.59, 31.08)  5.61  (4.09, 7.68) | 32.37  (31.62, 33.12)  17.99  (12.65, 25.59) | 34.85  (32.90, 36.80)  4.41  (3.06, 6.35) | 22.13  (21.84, 22.41)  74.59  (45.25, 122.94) |
| Nebraska | 28.04  (27.95, 28.14)  76.59  (72.72, 80.67) | -0.55 | 26.90  (26.61, 27.19)  94.77  (74.39, 120.73) | 32.83  (32.21, 33.44)  31.49  (25.63, 38.69) | 27.85  (27.56, 28.13)  92.08  (70.06, 121.02) | 33.64  (32.68, 34.59)  18.26  (15.27, 21.84) | 27.08  (26.55, 27.61)  41.95  (35.00, 50.28) | 25.59  (25.27, 25.91)  74.84  (55.41, 101.09) | 29.17  (28.91, 29.42)  109.59  (83.02, 144.67) | 33.27  (32.99, 33.55)  99.22  (77.57, 126.91) | 25.62  (25.50, 25.74)  325.34  (205.71, 514.55) | 29.55  (28.62, 30.47)  17.76  (14.55, 21.67) | 32.02  (31.62, 32.43)  64.20  (54.25, 75.98) | 30.44  (29.38, 31.51)  13.55  (10.80, 17.00) | 22.44  (22.32, 22.56)  363.38  (249.76, 528.68) |
| New Mexico | 28.06  (27.91, 28.21)  35.76  (33.43, 38.25) | -0.45 | 26.71  (26.26, 27.15)  44.04  (32.77, 59.18) | 31.07  (30.30, 31.84)  25.71  (21.75, 30.38) | 27.48  (27.12, 27.85)  59.28  (43.47, 80.84) | 32.47  (31.76, 33.17)  23.41  (18.36, 29.86) | 29.42  (28.65, 30.19)  22.29  (18.06, 27.50) | 24.74  (24.41, 25.06)  65.36  (43.51, 98.17) | 28.63  (28.25, 29.01)  48.60  (31.38, 75.29) | 30.92  (30.62, 31.23)  74.39  (52.26, 105.90) | 23.99  (23.68, 24.30)  74.32  (52.52, 105.17) | 26.25  (22.98, 29.51)  1.91  (1.20, 3.04) | 31.51  (30.88, 32.13)  27.14  (20.94, 35.18) | 28.65  (27.01, 30.29)  6.01  (4.35, 8.30) | 22.27  (22.00, 22.54)  85.71  (55.87, 131.49) |
| Oklahoma | 28.08  (27.97, 28.19)  56.05  (52.50, 59.84) | -0.41 | 26.42  (25.98, 26.86)  47.40  (36.79, 61.08) | 29.80  (29.24, 30.36)  36.13  (29.45, 44.34) | 26.71  (26.49, 26.93)  128.25  (90.24, 182.28) | 31.86  (30.64, 33.09)  11.29  (9.02, 14.14) | 29.50  (28.57, 30.43)  16.64  (13.35, 20.74) | 26.74  (26.31, 27.18)  47.74  (36.79, 61.95) | 29.28  (28.96, 29.61)  68.52  (48.79, 96.24) | 31.95  (31.56, 32.33)  59.35  (45.97, 76.61) | 26.43  (26.27, 26.59)  211.59  (141.93, 315.45) | 29.72  (28.83, 30.61)  15.58  (11.83, 20.52) | 32.10  (31.76, 32.45)  60.81  (41.75, 88.57) | 27.75  (26.55, 28.96)  9.85  (7.40, 13.10) | 22.35  (22.19, 22.52)  194.66  (131.52, 288.10) |
| Ohio | 28.24  (28.08, 28.39)  32.64  (30.58, 34.85) | -0.64 | 24.65  (24.23, 25.07)  49.03  (37.22, 64.57) | 33.16  (32.00, 34.32)  11.89  (9.43, 14.99) | 26.22  (25.86, 26.58)  58.36  (41.03, 83.03) | 33.86  (32.72, 34.99)  11.93  (9.37, 15.17) | 29.85  (28.09, 31.62)  7.66  (6.25, 9.39) | 25.32  (24.86, 25.78)  39.13  (27.71, 55.25) | 27.96  (27.45, 28.47)  35.02  (25.82, 47.50) | 32.65  (32.21, 33.10)  42.35  (30.52, 58.74) | 27.14  (26.81, 27.46)  66.72  (45.40, 98.04) | 30.00  (28.05, 31.96)  5.33  (4.04, 7.04) | 33.42  (32.79, 34.05)  26.29  (20.07, 34.45) | 30.74  (29.11, 32.26)  7.01  (5.39, 9.12) | 24.40  (24.20, 24.61)  157.28  (116.44, 212.46) |
| Idaho | 28.27  (28.15, 28.40)  52.91  (50.33, 55.63) | -0.13 | 26.64  (25.99, 27.29)  28.98  (23.58, 35.61) | 33.34  (32.79, 33.88)  36.70  (29.72, 45.31) | 24.78  (24.50, 25.06)  97.34  (74.49, 127.21) | 32.44  (28.00, 36.88)  2.14  (1.64, 2.80) | 23.07  (22.53, 23.61)  35.07  (27.44, 44.83) | 25.68  (25.27, 26.10)  59.63  (49.46, 71.88) | 29.09  (28.83, 29.36)  104.12  (78.01, 138.96) | 32.58  (32.40, 32.76)  195.53  (144.68, 264.25) | 25.97  (25.75, 26.18)  141.26  (104.65, 190.68) | 30.23  (29.29, 31.18)  13.69  (10.14, 18.47) | 32.97  (32.50, 33.43)  44.87  (35.60, 56.56) | 24.96  (23.31, 26.61)  7.28  (5.71, 9.28) | 20.90  (20.71, 21.10)  159.62  (112.81, 225.86) |
| Missouri | 28.36  (28.25, 28.47)  64.44  (61.07, 68.00) | -0.35 | 25.13  (24.71, 25.55)  53.98  (43.31, 67.30) | 30.91  (30.25, 31.57)  29.37  (24.27, 35.55) | 27.40  (27.13, 27.67)  100.37  (75.93, 132.67) | 33.92  (33.29, 34.55)  29.38  (23.64, 36.51) | 27.65  (26.86, 28.45)  24.22  (20.42, 28.72) | 25.25  (24.75, 25.75)  42.42  (34.37, 52.36) | 29.55  (29.27, 29.82)  100.15  (78.46, 127.82) | 33.20  (32.82, 33.57)  64.29  (51.67, 79.98) | 25.68  (25.54, 25.81)  260.86  (170.87, 398.23) | 30.90  (29.90, 31.91)  14.67  (11.69, 18.40) | 33.99  (33.63, 34.36)  61.98  (47.41, 81.04) | 28.45  (27.45, 29.45)  14.13  (11.09, 18.00) | 23.72  (23.59, 23.84)  316.81  (213.05, 471.12) |
| Arizona | 28.37  (28.13, 28.62)  14.79  (13.52, 16.19) | -0.69 | 26.99  (25.58, 28.39)  6.20  (3.98, 9.66) | 33.09  (30.88, 35.30)  4.46  (3.35, 5.95) | 27.30  (26.70, 27.90)  21.84  (13.03, 36.60) | 32.46  (26.98, 28.41)  1.53  (1.13, 2.07) | 27.62  (26.83, 28.41)  14.61  (9.12, 23.40) | 27.10  (26.41, 27.79)  20.19  (14.05, 29.01) | 27.29  (26.71, 27.86)  24.95  (16.23, 38.36) | 30.89  (30.39, 31.40)  29.50  (18.32, 47.50) | 25.45  (25.07, 25.83)  45.44  (26.59, 77.64) | 28.17  (26.24, 30.11)  3.60  (2.16, 5.99) | 32.69  (31.74, 33.63)  13.58  (10.02, 18.42) | 35.19  (31.73, 38.65)  3.10  (2.44, 3.94) | 23.47  (23.05, 23.89)  41.15  (26.23, 64.57) |
| Indiana | 28.38  (28.24, 28.53)  39.66  (37.54, 41.90) | -0.47 | 25.92  (25.53, 26.30)  56.48  (43.16, 73.92) | 31.79  (30.58, 33.00)  12.65  (10.41, 15.37) | 26.75  (26.36, 27.13)  59.18  (45.91, 76.29) | 32.93  (32.19, 33.67)  22.49  (17.81, 28.39) | 29.31  (27.97, 30.64)  10.82  (8.82, 13.26) | 25.38  (24.82, 25.95)  34.47  (27.60, 43.05) | 29.26  (28.77, 29.74)  42.78  (34.08, 53.69) | 33.46  (33.09, 33.84)  61.34  (48.02, 78.33) | 26.51  (26.22, 26.79)  91.05  (67.72, 122.40) | 29.58  (28.10, 31.06)  9.05  (7.29, 11.25) | 32.91  (32.21, 33.62)  25.90  (21.14, 31.73) | 31.89  (29.37, 34.42)  4.79  (3.85, 5.95) | 23.79  (23.62, 23.97)  201.20  (149.22, 271.29) |
| Illinois | 28.43  (28.29, 28.57)  42.81  (40.46, 45.30) | -0.29 | 25.80  (25.42, 26.19)  55.90  (42.29, 73.90) | 31.78  (30.83, 32.74)  15.72  (12.52, 19.73) | 27.60  (27.07, 28.12)  35.17  (26.84, 46.07) | 32.98  (32.25, 33.71)  22.75  (18.07, 28.64) | 28.83  (27.54, 30.12)  10.12  (7.98, 12.84) | 24.19  (23.54, 24.84)  28.17  (22.70, 34.96) | 29.78  (29.32, 30.24)  44.97  (35.33, 57.25) | 32.64  (32.23, 33.06)  55.59  (44.71, 69.12) | 25.69  (25.48, 25.91)  151.88  (116.11, 198.66) | 29.38  (28.11, 30.65)  11.71  (9.60, 14.28) | 32.50  (31.97, 33.04)  36.98  (29.46, 46.41) | 28.30  (26.64, 29.96)  8.56  (7.05, 10.40) | 24.55  (24.37, 24.74)  187.20  (142.43, 246.04) |
| District of Columbia | 28.44  (28.17, 28.72)  11.71  (10.63, 12.90) | 0.21 | 28.03  (27.09, 28.97)  11.07  (6.92, 17.70) | 35.25  (34.73, 35.76)  17.52  (2.52, 121.66) | 24.76  (22.56, 26.97)  2.51  (1.25, 5.05) | 26.86  (25.13, 28.59)  6.54  (5.06, 8.47) | 29.66  (27.48, 31.84)  2.53  (1.25, 5.13) | 24.13  (23.40, 24.86)  15.98  (9.50, 26.87) | 25.04  (23.88, 26.21)  9.53  (6.84, 13.27) | 31.48  (31.20, 31.76)  45.28  (4.56, 449.49) | 27.04  (26.71, 27.37)  50.05  (23.05, 108.70) | 32.16  (30.23, 34.08)  4.79  (3.44, 6.66) | 29.19  (28.12, 30.26)  10.74  (7.71, 14.96) | 14.50  (2.70, 26.30)  0.40  (0.23, 0.68) | 23.59  (23.12, 24.06)  41.49  (31.55, 54.54) |
| New Jersey | 28.64  (28.37, 28.90)  15.10  (14.10, 16.16) | -0.37 | 27.41  (26.82, 28.00)  26.43  (18.73, 37.29) | 34.13  (33.33, 34.94)  17.16  (12.66, 23.27) | 28.01  (25.97, 30.04)  5.34  (4.12, 6.92) | 28.68  (27.92, 29.45)  16.38  (10.95, 24.50) | 28.60  (26.72, 30.49)  4.69  (3.30, 6.69) | 24.64  (23.67, 25.62)  13.92  (10.69, 18.14) | 29.31  (28.31, 30.31)  13.58  (10.46, 17.64) | 29.53  (28.71, 30.34)  19.23  (15.18, 24.36) | 26.57  (26.08, 27.07)  42.50  (34.23, 52.76) | 27.32  (24.72, 29.92)  4.71  (3.81, 5.84) | 29.78  (28.36, 31.21)  8.89  (7.00, 11.28) | 35.74  (29.11, 42.37)  1.51  (1.18, 1.94) | 25.05  (24.57, 25.53)  40.40  (30.96, 52.73) |
| Georgia | 28.68  (28.56, 28.81)  47.76  (45.04, 50.65) | -0.54 | 25.00  (24.68, 25.32)  73.75  (54.97, 98.95) | 35.24  (34.74, 35.74)  39.99  (31.67, 50.49) | 27.73  (27.52, 27.94)  144.92  (104.17, 201.61) | 30.22  (28.99, 31.46)  11.76  (9.55, 14.48) | 27.53  (27.01, 28.04)  37.48  (29.35, 47.87) | 26.27  (25.83, 26.71)  46.60  (35.86, 60.56) | 27.89  (27.45, 28.33)  46.27  (35.24, 60.76) | 33.17  (32.79, 33.56)  62.71  (50.52, 77.84) | 27.20  (26.93, 27.47)  97.85  (72.84, 131.44) | 27.89  (27.02, 28.76)  16.68  (12.90, 21.56) | 32.25  (31.63, 32.88)  30.18  (24.42, 37.28) | 29.94  (28.31, 31.56)  8.04  (6.46, 10.00) | 24.17  (23.96, 24.38)  134.63  (91.88, 197.28) |
| Montana | 28.70  (28.62, 28.78)  121.72  (117.38, 126.22) | -0.46 | 27.00  (26.66, 27.34)  95.00  (83.44, 108.15) | 35.79  (35.39, 36.19)  75.19  (66.03, 85.63) | 26.55  (26.39, 26.71)  255.54  (206.58, 316.11) | 37.30  (36.65, 37.95)  32.76  (27.74, 38.69) | 25.06  (24.54, 25.57)  42.35  (35.00, 51.25) | 23.71  (23.38, 24.04)  90.99  (77.66, 106.59) | 30.41  (30.31, 30.50)  559.70  (403.61, 776.15) | 32.61  (32.42, 32.81)  207.76  (176.85, 244.06) | 24.27  (24.17, 24.38)  501.76  (382.04, 659.01) | 27.15  (26.39, 27.91)  24.34  (20.18, 29.35) | 32.48  (32.11, 32.85)  72.04  (59.76, 86.83) | 35.30  (34.60, 36.00)  27.32  (22.62, 33.00) | 22.69  (22.55, 22.83)  292.86  (215.47, 398.05) |
| Arkansas | 28.70  (28.59, 28.80)  65.38  (61.61, 69.38) | -0.28 | 25.63  (25.26, 26.01)  61.63  (47.82, 79.45) | 32.21  (31.85, 32.57)  63.54  (48.66, 82.98) | 27.49  (27.30, 27.69)  147.16  (98.61, 219.61) | 34.07  (33.16, 34.98)  15.99  (12.47, 20.49) | 28.19  (27.62, 28.75)  36.74  (30.22, 44.66) | 28.45  (27.94, 28.96)  36.69  (28.02, 48.06) | 29.66  (29.45, 29.87)  135.95  (94.31, 195.96) | 33.78  (33.41, 34.15)  62.83  (49.27, 80.12) | 26.13  (25.97, 26.29)  228.16  (165.70, 314.17) | 28.31  (27.48, 29.14)  19.20  (15.30, 24.08) | 33.82  (33.48, 34.15)  63.79  (43.75, 93.03) | 26.62  (25.24, 28.00)  8.95  (6.96, 11.50) | 22.80  (22.65, 22.96)  222.51  (146.33, 338.35) |
| Alabama | 28.70  (28.59, 28.82)  58.54  (55.47, 61.79) | -0.56 | 23.57  (23.23, 23.90)  84.86  (70.95, 101.50) | 33.65  (33.20, 34.11)  50.55  (41.60, 61.43) | 27.14  (26.92, 27.37)  139.42  (105.52, 184.21) | 32.11  (31.10, 33.13)  15.04  (12.16, 18.59) | 27.32  (26.77, 27.88)  34.07  (26.83, 43.27) | 27.73  (27.30, 28.17)  50.98  (40.63, 63.97) | 29.21  (28.89, 29.54)  74.24  (56.05, 98.33) | 33.65  (33.32., 33.98)  78.93  (62.86, 99.11) | 26.70  (26.52, 26.88)  178.78  (125.89, 253.89) | 29.22  (28.46, 29.97)  21.19  (16.58, 27.06) | 31.96  (31.59, 32.32)  59.72  (44.16, 80.75) | 29.02  (27.96, 30.08)  12.59  (9.75, 16.27) | 23.63  (23.42, 23.83)  142.11  (97.02, 208.17) |
| Tennessee | 28.73  (28.62, 28.83)  63.59  (60.16, 67.23) | -0.42 | 24.59  (24.29, 24.90)  82.66  (63.29, 107.96) | 33.00  (32.47, 33.53)  38.13  (30.61, 47.49) | 26.75  (26.50, 26.99)  106.90  (73.88, 154.69) | 33.57  (32.84, 34.30)  23.10  (18.47, 28.91) | 28.65  (27.98, 29.33)  26.70  (21.52, 33.13) | 25.62  (25.23, 26.00)  57.32  (44.20, 74.32) | 26.98  (26.77, 27.19)  144.56  (105.86, 197.39) | 34.38  (34.13, 34.63)  107.84  (78.00, 149.10) | 27.32  (27.11, 27.52)  150.63  (108.63, 208.87) | 30.17  (29.55, 30.79)  27.27  (20.90, 35.57) | 33.88  (33.54, 34.21)  69.52  (51.94, 93.05) | 30.04  (28.52, 31.55)  7.89  (6.13, 10.16) | 23.46  (23.31, 23.60)  250.16  (164.43, 380.57) |
| Maryland | 28.77  (28.55, 28.98)  19.07  (17.74, 20.50) | -0.45 | 27.04  (26.51, 27.58)  27.17  (16.78, 44.01) | 34.44  (33.46, 35.42)  12.71  (16.78, 44.01) | 27.96  (26.92, 29.00)  10.51  (7.22, 15.29) | 29.78  (28.70, 30.87)  11.68  (8.85, 15.41) | 34.04  (30.86, 37.21)  3.49  (2.77, 4.41) | 24.28  (23.51, 25.06)  22.04  (17.89, 27.16) | 29.64  (28.81, 30.46)  18.26  (14.23, 23.42) | 32.51  (31.98, 33.04)  32.62  (23.96, 44.40) | 26.80  (26.30, 27.31)  35.88  (26.78, 48.08) | 26.88  (24.05, 29.72)  3.40  (2.57, 4.48) | 31.02  (30.38, 31.66)  20.47  (12.95, 32.33) | 31.92  (29.12, 34.73)  2.89  (2.04, 4.07) | 24.27  (24.00, 24.53)  94.57  (65.93, 135.64) |
| Iowa | 28.80  (28.70, 28.90)  75.09  (71.40, 78.97) | -0.53 | 25.84  (25.49, 26.19)  73.11  (58.89, 90.75) | 34.47  (33.81, 35.12)  28.82  (23.51, 35.33) | 26.80  (26.59, 27.00)  146.64  (102.48, 209.82) | 32.82  (32.10, 33.55)  24.89  (20.41, 30.35) | 27.03  (26.59, 27.48)  49.67  (39.97, 61.73 | 27.08  (26.61, 27.55)  47.97  (39.39, 58.43) | 29.68  (29.48, 29.89)  151.44  (110.62, 207.32) | 33.65  (33.38, 33.92)  113.51  (92.25, 139.69) | 25.46  (25.31, 25.60)  275.07  (205.94, 367.41) | 30.44  (29.29, 31.59)  14.47  (12.12, 17.27) | 33.64  (33.29, 33.98)  73.17  (58.27, 91.88) | 30.84  (29.85, 31.82)  14.93  (11.87, 18.78) | 23.97  (23.83, 24.10)  270.76  (178.13, 411.57) |
| Mississippi | 28.85  (28.77, 28.93)  102.69  (98.06, 107.53) | -0.57 | 22.60  (22.42, 22.79)  183.36  (139.63, 240.80) | 32.67  (32.45, 32.89)  145.22  (112.35, 187.72) | 27.87  (27.70, 28.04)  227.24  (177.06, 291.63) | 33.25  (32.61, 33.88)  31.80  (26.46, 38.22) | 27.60  (27.36, 27.84)  121.66  (92.00, 160.87) | 28.98  (28.60, 29.36)  64.43  (52.48, 79.10) | 27.99  (27.78, 28.19)  151.56  (113.99, 201.51) | 33.78  (33.56, 33.99)  160.41  (128.47, 200.29) | 26.40  (26.28, 26.52)  342.13  (233.17, 502.00) | 28.82  (28.34, 29.31)  38.80  (29.10, 51.72) | 33.43  (33.17, 33.69)  111.11  (86.56, 142.63) | 29.59  (28.66, 30.52)  17.80  (14.60, 21.70) | 22.77  (22.58, 22.96)  178.69  (135.28, 236.03) |
| Pennsylvania | 28.86  (28.69, 29.03)  27.01  (25.12, 29.04) | -0.67 | 26.80  (26.34, 27.25)  44.33  (34.10, 57,62) | 34.65  (33.71, 35.59)  16.06  (12.80, 20.15) | 27.79  (26.94, 28.64)  17.02  (13.05, 22.19) | 32.77  (31.74, 33.80)  12.67  (9.64, 16.66) | 29.66  (27.89, 31.43)  6.15  (4.70, 8.06) | 24.69  (24.16, 25.21)  36.38  (28.27, 46.83) | 29.53  (29.06, 30.01)  37.25  (26.60, 52.16) | 31.68  (31.18, 32.18)  34.81  (24.83, 48.80) | 26.89  (26.56, 27.22)  66.36  (45.55, 96.67) | 29.94  (28.11, 31.78)  5.74  (4.34, 7.58) | 31.94  (31.31, 32.57)  23.66  (16.81, 33.30) | 31.89  (29.46, 34.32)  3.44  (2.44, 4.86) | 24.76  (24.53, 24.98)  114.81  (72.90, 180.82) |
| North Dakota | 28.94  (28.85, 29.02)  102.19  (98.17, 106.38) | -0.45 | 26.48  (26.29, 26.68)  205.00  (172.07, 244.23) | 34.64  (34.14, 35.15)  55.35  (48.87, 62.70) | 27.58  (27.34, 27.82)  129.80  (103.13, 163.36) | 32.59  (31.89, 33.28)  30.19  (25.69, 35.47) | 27.95  (27.33, 28.56)  38.97  (33.93, 44.77) | 23.38  (23.09, 23.67)  93.64  (72.82, 120.41) | 30.72  (30.59, 30.85)  328.32  (244.63, 440.65) | 31.27  (31.00, 31.54)  126.88  (107.96, 149.12) | 24.92  (24.84, 25.00)  591.24  (319.95, 1092.58) | 29.43  (28.68, 30.19)  24.68  (20.48, 29.75) | 34.49  (34.22, 24.76)  99.31  (73.14, 134.86) | 36.92  (36.46, 37.39)  48.36  (39.64, 59.00) | 23.44  (23.26, 23.63)  184.48  (140.41, 242.38) |
| Virginia | 28.96  (28.83, 29.09)  45.58  (42.69, 48.67) | -0.50 | 25.87  (25.52, 26.21)  59.84  (40.95, 87.43) | 35.82  (35.14, 36.50)  22.95  (17.25, 30.55) | 27.98  (27.54, 28.41)  43.49  (31.28, 60.45) | 31.92  (31.21, 32.62)  24.00  (18.98, 30.35) | 28.89  (27.85, 29.93)  11.49  (8.35, 15.81) | 25.19  (24.82, 25.57)  58.02  (42.86, 78.55) | 29.23  (28.80, 29.65)  46.36  (33.98, 63.25) | 32.74  (32.45, 33.02)  84.24  (59.12, 120.02) | 28.00  (27.70, 28.29)  78.11  (53.10, 114.90) | 29.25  (28.23, 30.26)  12.75  (9.64, 16.86) | 32.71  (32.29, 33.12)  48.36  (35.86, 65.23) | 33.27  (30.50, 36.03)  4.10  (3.25, 5.16) | 24.21  (24.06, 24.36)  247.87  (182.62, 336.42) |
| Delaware | 29.00  (28.71, 29.30)  15.00  (14.20, 15.84) | -0.46 | 26.20  (25.60, 26.80)  29.57  (23.07, 37.90) | 34.36  (31.79, 36.93)  4.90  (3.98, 6.03) | 31.17  (29.71, 32.63)  8.04  (6.18, 10.45) | 31.00  (29.88, 32.11)  12.77  (10.19, 16.01) | 25.21  (23.20, 27.22)  6.06  (4.85, 7.59) | 22.02  (21.00, 23.04)  13.62  (10.63, 17.45) | 28.73  (27.94, 29.51)  20.78  (16.59, 26.02) | 29.94  (29.05, 30.82)  22.63  (19.44, 26.33) | 27.32  (26.75, 27.89)  29.67  (21.98, 40.06) | 28.83  (25.77, 31.89)  4.31  (3.55, 5.23) | 31.41  (30.69, 32.13)  26.45  (21.99, 31.82) | 35.26  (32.39, 38.14)  5.24  (4.43, 6.20) | 24.92  (24.44, 25.41)  37.49  (27.23, 51.62) |
| Michigan | 29.03  (28.85, 29.20)  27.63  (25.78, 29.62) | -0.42 | 26.34  (25.73, 26.95)  26.46  (19.49, 35.92) | 33.76  (32.72, 34.80)  12.03  (9.01, 16.06) | 26.95  (26.46, 27.45)  35.95  (26.21, 49.31) | 34.09  (32.93, 35.26)  11.23  (8.75, 14.41) | 28.41  (27.01, 29.81)  8.49  (6.52, 11.06) | 24.86  (24.20, 25.51)  24.79  (18.82, 32.66) | 30.53  (30.14, 30.93)  51.85  (37.56, 71.58) | 33.42  (32.98, 33.86)  42.13  (29.75, 59.68) | 25.94  (25.65, 26.23)  80.65  (55.93, 116.29) | 29.20  (27.59, 30.81)  6.29  (4.61, 8.60) | 34.64  (33.96, 35.31)  22.05  (16.11, 30.18) | 29.35  (27.48, 31.22)  5.78  (4.42, 7.55) | 24.57  (24.35, 24.80)  117.54  (79.06, 174.75) |
| North Carolina | 29.05  (28.90, 29.19)  37.54  (35.17, 40.06) | -0.46 | 25.78  (25.39, 26.17)  48.06  (32.08, 71.99) | 35.19  (34.71, 35.68)  36.53  (26.34, 50.66) | 28.59  (28.08., 29.10)  32.07  (21.78, 47.23) | 32.72  (32.12, 33.32)  27.82  (20.97, 36.91) | 30.11  (29.16, 31.06)  14.12  (10.73, 18.59) | 24.96  (24.47, 25.44)  39.26  (29.89, 51.55) | 29.33  (28.85, 29.80)  38.90  (28.70, 52.72) | 32.91  (32.52, 33.30)  54.62  (41.30, 72.23) | 26.90  (26.62, 27.18)  92.35  (68.66, 124.22) | 29.60  (28.34, 30.87)  9.83  (7.57, 12.76) | 33.17  (32.61, 33.74)  33.09  (25.99, 42.12) | 31.36  (29.72, 32.99)  6.80  (5.19, 8.91) | 23.56  (23.40, 23.71)  197.30  (120.61, 322.75) |
| Connecticut | 29.09  (28.92, 29.27)  27.92  (26.12, 29.83) | -0.44 | 27.18  (26.69, 27.67)  31.82  (20.43, 49.57) | 34.36  (33.29, 35.43)  10.12  (6.96, 14.71) | 29.00  (27.68, 30.32)  8.25  (6.04, 11.26) | 30.30  (28.88, 31.73)  7.94  (6.00, 10.52) | 29.68  (28.09, 31.27)  6.08  (4.34, 8.52) | 24.05  (23.11, 24.99)  14.67  (11.26, 19.12) | 29.63  (29.02, 30.25)  27.08  (20.45, 35.85) | 30.70  (30.32, 31.09)  60.31  (47.64, 76.35) | 27.16  (26.81, 27.50)  75.32  (60.78, 93.34) | 29.71  (27.92, 31.49)  7.63  (6.23, 9.33) | 30.27  (29.85, 30.69)  50.93  (39.13, 66.29) | 31.33  (28.80, 33.86)  4.89  (3.95, 6.04) | 24.68  (24.39, 24.96)  99.54  (78.58, 126.10) |
| New York | 29.10  (28.88, 29.32)  17.11  (15.67, 18.68) | -0.24 | 26.25  (25.58, 26.93)  22.04  (16.05, 30.26) | 33.47  (32.57, 34.36)  14.66  (10.84, 19.82) | 28.00  (26.75, 29.26)  9.25  (6.91, 12.39) | 29.12  (28.01, 30.23)  10.63  (7.82, 14.47) | 28.74  (27.02, 30.47)  5.33  (3.76, 7.56) | 24.77  (23.95, 25.60)  15.75  (11.21, 22.12) | 29.47  (28.92, 30.02)  28.78  (20.16, 41.08) | 30.77  (30.29, 31.24)  35.99  (24.88, 52.04) | 26.64  (26.28, 27.00)  50.43  (29.96, 84.86) | 28.12  (26.01, 30.23)  4.02  (2.81, 5.75) | 32.53  (31.85, 33.22)  19.04  (12.32, 29.42) | 30.49  (26.09, 34.89)  1.65  (1.15, 2.36) | 24.51  (24.07, 24.96)  38.02  (24.53, 58.94) |
| Minnesota | 29.12  (29.00, 29.24)  61.04  (58.44, 63.76) | -0.50 | 26.02  (25.71, 26.33)  88.84  (71.28, 110.72) | 34.21  (33.63, 34.79)  37.95  (31.98, 45.05) | 27.18  (26.83, 27.52)  85.47  (72.82, 100.32) | 32.52  (31.32, 33.72)  15.43  (13.25, 17.96) | 26.51  (25.54, 27.47)  20.57  (17.72, 23.88) | 25.44  (24.88, 26.01)  38.30  (32.01, 45.82) | 29.72  (29.51, 29.94)  155.46  (122.84, 196.73) | 32.63  (32.31, 32.96)  85.30  (70.13, 103.75) | 25.33  (25.15, 25.51)  203.74  (157.68, 263.25) | 30.85  (29.09, 32.61)  8.40  (6.99, 10.09) | 33.85  (33.42, 34.28)  56.50  (47.20, 67.64) | 32.98  (32.35, 33.61)  31.12  (25.70, 37.68) | 24.71  (24.50, 24.93)  148.23  (112.98, 194.48) |
| Wisconsin | 29.17  (29.07, 29.27)  68.97  (65.11, 73.06) | -0.34 | 26.78  (26.45, 27.11)  69.75  (51.15, 95.12) | 32.84  (32.18, 33.50)  28.08  (22.79, 34.59) | 26.96  (26.66, 27.26)  79.00  (56.27, 110.91) | 32.31  (31.44, 33.19)  18.12  (14.54, 22.57) | 28.13  (27.13, 29.12)  15.80  (12.87, 19.41) | 24.32  (23.99, 24.65)  72.85  (54.87, 96.73) | 31.11  (30.91, 31.31)  146.15  (100.19, 213.18) | 33.40  (33.18, 33.62)  147.91  (114.26, 191.47) | 25.62  (25.49, 25.74)  323.15  (22.17, 470.03) | 27.25  (25.93, 28.57)  9.19  (7.07, 11.95) | 34.01  (33.67, 34.34)  65.54  (46.60, 92.19) | 32.18  (21.32, 33.04)  16.73  (12.86, 21.75) | 24.67  (24.52, 24.81)  235.10  (145.81, 379.09) |
| Kentucky | 29.27  (29.15, 29.38)  58.86  (55.97, 61.91) | -0.48 | 25.13  (24.91, 25.36)  132.37  (99.19, 176.66) | 32.86  (32.28, 33.44)  36.11  (30.11, 43.29) | 27.23  (26.92, 27.54)  88.67  (71.57, 109.85) | 34.11  (33.72, 34.49)  61.66  (49.58, 76.69) | 30.52  (29.75, 31.29)  24.06  (19.99, 28.96) | 26.16  (25.74, 26.59)  57.62  (47.74, 69.55) | 28.21  (27.91, 28.50)  92.62  (73.23, 117.15) | 33.58  (33.34, 33.82)  121.83  (93.50, 158.74) | 27.12  (26.91, 27.34)  128.18  (86.08, 190.87) | 29.25  (28.32, 30.18)  17.61  (14.39, 21.55) | 33.46  (33.03, 33.88)  48.71  (37.17, 63.83) | 28.52  (26.98, 30.07)  8.50  (6.82, 10.59) | 23.74  (23.53, 23.95)  140.43  (98.86, 199.47) |
| West Virginia | 29.28  (29.19, 29.37)  94.61  (90.66, 98.73) | -0.37 | 26.79  (26.55, 27.03)  135.01  (108.63, 167.79) | 34.65  (34.05, 35.25)  37.59  (32.03, 44.11) | 27.85  (27.60, 28.09)  138.98  (117.38, 164.54) | 31.59  (31.18, 32.00)  69.37  (60.28, 79.84) | 29.03  (28.43, 29.64)  39.46  (34.26, 45.45) | 25.64  (25.38, 25.90)  127.96  (107.00, 153.02) | 30.11  (29.89, 30.33)  149.84  (118.34, 189.72) | 32.90  (32.74, 33.06)  235.36  (174.70, 317.08) | 27.55  (27.36, 27.74)  183.42  (142.30, 236.42) | 29.70  (29.12, 30.27)  36.08  (29.84, 43.62) | 34.02  (33.73, 34.31)  91.04  (69.04, 120.04) | 29.86  (28.41, 31.31)  10.04  (8.25, 12.21) | 22.66  (22.53, 22.78)  322.48  (228.92, 454.28) |
| South Dakota | 29.37  (29.31, 29.43)  197.39  (191.41, 203.55) | -0.47 | 25.74  (25.55, 25.93)  214.97  (184.40, 250.62) | 35.09  (34.70, 35.49)  75.34  (65.96, 86.05) | 27.59  (27.38, 27.80)  204.24  (180.71, 230.84) | 31.36  (30.69, 32.03)  39.31  (35.01, 44.14) | 29.74  (29.32, 30.17)  75.69  (67.78, 84.53) | 25.16  (24.92, 25.41)  153.12  (132.66, 176.73) | 29.35  (29.23, 29.48)  419.93  (349.58, 504.44) | 32.76  (32.63, 32.89)  431.38  (373.30, 498.51) | 25.53  (25.45, 25.61)  860.99  (717.37, 1033.37) | 31.59  (31.12, 32.05)  54.22  (46.25, 63.57) | 33.27  (33.15, 33.38)  426.80  (326.27, 558.32) | 35.95  (35.59, 36.30)  70.97  (56.97, 88.41) | 23.22  (23.10, 23.33)  436.14  (334.02, 569.47) |
| South Carolina | 29.41  (29.25, 29.58)  30.62  (28.56, 32.83) | 0.06 | 25.96  (25.34, 26.58)  26.29  (19.56, 35.33) | 35.13  (34.73, 35.53)  48.27  (33.17, 70.25) | 28.57  (27.99, 29.15)  26.24  (18.13, 37.97) | 33.49  (32.95, 34.04)  29.97  (21.33, 42.10) | 28.73  (28.02, 29.44)  22.83  (17.77, 29.33) | 26.44  (25.69, 27.20)  20.47  (15.78, 26.54) | 28.43  (27.79, 29.07)  27.88  (22.18, 35.04) | 32.12  (31.58, 32.67)  34.85  (27.51, 44.17) | 27.12  (26.79, 27.45)  67.72  (48.34, 94.85) | 29.28  (27.77, 30.80)  7.46  (5.68, 9.80) | 32.15  (31.56, 32.73)  26.95  (19.20, 37.83) | 27.87  (25.72, 30.02)  4.70  (3.56, 6.21) | 24.20  (24.03, 24.37)  161.49  (88.55, 294.52) |
| Massachusetts | 29.43  (29.24, 29.63)  23.91  (22.45, 25.46) | -0.45 | 27.85  (27.35, 28.35)  35.99  (26.41, 49.03) | 34.68  (33.96, 35.40)  23.15  (18.31, 29.27) | 27.30  (25.82, 28.78)  8.59  (6.80, 10.84) | 32.26  (31.10, 33.42)  11.03  (8.50, 14.31) | 30.17  (28.79, 31.55)  9.07  (7.08, 11.61) | 25.14  (24.39, 25.89)  23.03  (18.63, 28.47) | 30.17  (28.79, 31.55)  35.94  (26.66, 48.44) | 31.63  (31.10, 32.15)  35.60  (27.53, 46.05) | 26.60  (26.18, 27.01)  48.55  (35.81, 65.81) | 29.19  (27.11, 31.26)  5.96  (4.79, 7.42) | 32.72  (31.84, 33.61)  16.70  (13.05, 21.39) | 34.76  (29.84, 39.67)  2.18  (1.72, 2.75) | 25.30  (25.03, 25.56)  104.21  (78.22, 138.83) |
| Vermont | 29.91  (29.76, 30.05)  35.04  (32.46, 37.82) | -0.14 | 26.98  (25.94, 28.02)  10.43  (7.12, 15.26) | 36.75  (36.26, 37.25)  30.50  (18.99, 48.99) | 27.63  (27.00, 28.26)  23.12  (15.97, 33.46) | 34.63  (34.03, 35.22)  20.19  (10.41, 39.18) | 30.04  (29.52, 30.57)  18.81  (4.62, 76.66) | 26.35  (26.07, 26.64)  93.81  (71.57, 122.97) | 30.26  (29.87, 30.64)  53.93  (39.27, 74.07) | 31.49  (31.23, 31.76)  79.19  (43.21, 145.15) | 27.17  (26.92, 27.43)  94.63  (63.55, 140.91) | 31.52  (29.99, 33.04)  9.79  (8.13, 11.79) | 33.77  (33.45, 34.08)  49.01  (17.10, 140.46) | 26.15  (24.34, 27.96)  6.90  (5.52, 8.64) | 25.21  (24.92, 25.50)  85.35  (62.63, 116.31) |
| Rhode Island | 29.93  (29.66, 30.19)  15.44  (14.44, 16.50) | -0.29 | 29.60  (28.62, 30.58)  14.82  (11.68, 18.81) | 35.83  (35.02, 36.64)  20.66  (16.70, 25.55) | 31.23  (29.14, 33.31)  4.63  (3.43, 6.24) | 31.93  (29.73, 34.13)  4.63  (3.52, 6.10) | 30.49  (28.47, 32.50)  5.97  (4.75, 7.51) | 24.53  (23.71, 25.34)  19.12  (15.08, 24.25) | 29.99  (29.38, 30.59)  31.69  (25.75, 39.00) | 31.78  (30.82, 32.74)  16.30  (13.19, 20.15) | 26.69  (26.23, 27.14)  42.20  (31.04, 57.36) | 36.56  (33.73, 39.39)  3.15  (2.33, 4.28) | 32.16  (31.90, 32.41)  47.32  (2.11, 1063.14) | 29.01  (23.99, 34.04)  1.55  (1.12, 2.15) | 24.48  (24.08, 24.87)  47.92  (32.28, 71.13) |
| New Hampshire | 29.99  (29.82, 30.16)  29.91  (28.08, 31.87) | -0.41 | 28.16  (27.65, 28.67)  35.34  (26.44, 47.24) | 33.97  (33.32, 34.61)  21.63  (14.49, 32.30) | 30.11  (28.24, 31.99)  6.00  (4.66, 7.73) | 32.58  (31.50, 33.67)  11.17  (8.25, 15.11) | 33.16  (30.73, 35.59)  5.13  (4.15, 6.34) | 25.03  (24.47, 25.59)  32.55  (25.24, 41.96) | 31.41  (31.08, 31.73)  72.06  (53.01, 97.95) | 31.66  (31.03, 32.30)  30.87  (25.47, 37.41) | 26.93  (26.72, 27.14)  125.11  (74.28, 210.74) | 30.02  (28.01, 32.02)  6.08  (4.85, 7.61) | 33.38  (32.84, 33.93)  35.61  (28.31, 44.79) | 31.81  (30.30, 33.31)  9.10  (7.38, 11.22) | 25.38  (25.09, 25.67)  87.71  (65.26, 117.89) |
| Maine | 30.36  (30.26, 30.46)  71.23  (67.38, 75.31) | -0.47 | 27.54  (27.23, 27.84)  90.57  (72.95, 112.44) | 35.47  (34.86, 36.08)  29.25  (22.92, 37.34) | 27.29  (26.83, 27.75)  45.11  (35.34, 57.58) | 32.78  (32.07, 33.49)  22.68  (17.53, 29.34) | 29.81  (29.22, 30.41)  31.97  (25.79, 39.63) | 27.04  (26.45, 27.62)  36.31  (30.29, 43.52) | 31.42  (31.28, 31.55)  310.24  (234.58, 410.29) | 33.21  (32.93, 33.48)  106.31  (84.76, 133.33) | 26.78  (26.62, 26.95)  205.76  (144.99, 292.00) | 32.19  (30.79, 33.59)  9.66  (7.76, 12.02) | 33.41  (32.96, 33.87)  42.26  (31.58, 56.55) | 32.10  (31.24, 32.97)  17.25  (13.48, 22.06) | 25.61  (25.42, 25.80)  189.00  (148.69, 240.23) |

*Spearman rho correlation coefficient

Table S1. Peak timing and absolute intensity and their 95% CIs, and Spearman correlations for each of the 48 states both seasonally and overall (Model 3) in ascending order of the average peak week of the 13 influenza seasons. For each cell, peak timing is on the first line, 95% CI for peak timing is on the second line, absolute intensity is on the third line, and 95% CI for absolute intensity is on the fourth line. Spearman correlations represent the association between peak timing and absolute intensity for each state for all 13 seasons.
